# β-Arrestin Based Receptor Signaling Paradigms: Potential Therapeutic Targets for Complex Age-Related Disorders

**DOI:** 10.3389/fphar.2018.01369

**Published:** 2018-11-28

**Authors:** Jaana van Gastel, Jhana O. Hendrickx, Hanne Leysen, Paula Santos-Otte, Louis M. Luttrell, Bronwen Martin, Stuart Maudsley

**Affiliations:** ^1^Department of Biomedical Sciences, University of Antwerp, Antwerp, Belgium; ^2^Translational Neurobiology Group, Centre for Molecular Neuroscience, VIB, Antwerp, Belgium; ^3^Institute of Biophysics, Humboldt University of Berlin, Berlin, Germany; ^4^Division of Endocrinology, Diabetes and Medical Genetics, Medical University of South Carolina, Charleston, SC, United States

**Keywords:** β-arrestin signaling, ligand ‘bias’, GPCRs, age-related disorders, precision, tailored efficacy

## Abstract

G protein coupled receptors (GPCRs) were first characterized as signal transducers that elicit downstream effects through modulation of guanine (G) nucleotide-binding proteins. The pharmacotherapeutic exploitation of this signaling paradigm has created a drug-based field covering nearly 50% of the current pharmacopeia. Since the groundbreaking discoveries of the late 1990s to the present day, it is now clear however that GPCRs can also generate productive signaling cascades through the modulation of β-arrestin functionality. β-Arrestins were first thought to only regulate receptor desensitization and internalization – exemplified by the action of visual arrestin with respect to rhodopsin desensitization. Nearly 20 years ago, it was found that rather than controlling GPCR signal termination, productive β-arrestin dependent GPCR signaling paradigms were highly dependent on multi-protein complex formation and generated long-lasting cellular effects, in contrast to G protein signaling which is transient and functions through soluble second messenger systems. β-Arrestin signaling was then first shown to activate mitogen activated protein kinase signaling in a G protein-independent manner and eventually initiate protein transcription – thus controlling expression patterns of downstream proteins. While the possibility of developing β-arrestin biased or functionally selective ligands is now being investigated, no additional research has been performed on its possible contextual specificity in treating age-related disorders. The ability of β-arrestin-dependent signaling to control complex and multidimensional protein expression patterns makes this therapeutic strategy feasible, as treating complex age-related disorders will likely require therapeutics that can exert network-level efficacy profiles. It is our understanding that therapeutically targeting G protein-independent effectors such as β-arrestin will aid in the development of precision medicines with tailored efficacy profiles for disease/age-specific contextualities.

## Introduction

The β-arrestin family comprises four members: visual arrestins (arrestin1 and arrestin4) and the non-visual arrestins (β-arrestin1 and β-arrestin2, also referred to as arrestin2 and arrestin3, respectively). Furthermore, an α-arrestin family, structurally related to the β-arrestins has been identified (Chutkow et al., 2010). To provide a more therapeutically-targeted discussion in this review we will focus on the β-arrestins 1 and -2, which are ubiquitously expressed in most mammalian tissues and cell types (Feng et al., 2011). Heptahelical transmembrane GPCRs, were originally thought to be a member of a three-part functional signaling system: (1) the receptor detecting ligands in the extracellular milieu, (2) a heterotrimeric (αβγ) G protein that dissociates into α-subunits bound to guanosine triphosphate (GTP) and βγ-subunits, after interaction with the ligand-bound GPCR, and (3) an effector interacting with the dissociated G protein subunits (α or βγ) to generate soluble small molecule second messengers. This GPCR-G protein interaction is catalytic, i.e., one receptor can sequentially activate multiple G proteins either of the same or of a different type. GPCR signaling through G proteins is terminated after serine-threonine phosphorylation of the receptors, both by members of the GPCR kinase family and second messenger-activated protein kinases such as protein kinase A and C. This is followed by arrestin binding (β-arrestins1 or 2), which sterically inhibits further G protein activation (Freedman and Lefkowitz, 1996). Next, the ligand-bound receptor can be internalized through an arrestin-dependent engagement of clathrin-coated pits (Ferguson et al., 1996; Goodman et al., 1996), after which the receptors are either dephosphorylated and recycled back to the cell surface or targeted for lysosomal degradation if a potent ligand stimulation level persists (Yu et al., 1993).

While this canonical description of GPCR signaling still largely holds true, this is not where GPCR signaling ends. Luttrell et al. (1999) discovered that β-arrestin becomes part of a multi-protein signaling complex (often termed a ‘*receptorsome*’) which functions both to target the receptor-kinase complex to clathrin-coated pits and additionally to recruit activated non-receptor tyrosine kinase c-Src to the plasma membrane. This ‘*receptorsome’* formation process engenders a receptor-based capacity to induce a novel signaling cascade distinct from the G protein-dependent paradigm. Thus, it was elegantly demonstrated that β-arrestins did not only ‘arrest’ G protein signaling, but transformed the signaling activity and initiated supplementary signaling cascades where the *‘desensitized’* receptor functioned as a part of a mitogenic signaling complex (Luttrell et al., 1999). Additionally, while G protein-dependent signaling is essentially a transient catalytic process which generates soluble small molecule second messenger products, it is evident that β-arrestin-dependent signaling can engender highly characteristic and translatable transcriptomic phenotypes, presumably via the formation of more self-reinforced, complex, higher-order multi-protein signaling interactomes (Maudsley et al., 2015, 2016) similar to the *‘encryptome’* complexes proposed for growth factor receptor signaling (Martin et al., 2009). This GPCR interactome capacity for regulating complex transcription patterns, thus revealed the ability for β-arrestin-dependent signaling to create discrete, reinforced coherent patterns of long-term signal transduction. This new signaling paradigm engenders a feasible mechanism for receptor-based cell/tissue engineering independent of traditional GPCR signaling modalities. Since this discovery, considerable research has been undertaken to further investigate the functional role(s) of β-arrestin, beyond its function in receptor desensitization, and has thus rekindled the concept of ‘Agonist Trafficking’ in the new form of drug-focused *‘Biased’* signaling (Kenakin, 1995; Andresen and Luttrell, 2011).

### GPCR Biased Signaling

The past two decades of GPCR biology research has reshaped the world of receptor signaling and pharmacotherapeutics via the maturation of two critical concepts: (1) receptor signaling pluridimensionality (Maudsley et al., 2005) and (2) ligand bias (Luttrell and Kenakin, 2011). The effective exploitation of these two facets of receptor functionality will likely facilitate the development in the future of more effective GPCR-based therapeutics (i.e., higher functional specificity and reduced ‘off-target’ actions) (Luttrell and Kenakin, 2011). Pluridimensional efficacy of GPCRs refers to the discovery that these receptors signal via multiple G protein and non-G protein effectors, and can thus adopt multiple ‘active’ states. Essentially, this also introduces the concept that a GPCR is never truly quiescent and simply exists in different proportions of various active state complexes in distinct cellular sub-compartments. The stable interactions between GPCRs and β-arrestin enable the formation of receptor-based signal-specific interactomes. Here we can potentially term these structures *‘encryptomes’* – the specific protein stoichiometry essentially ‘encrypts’ the specificity of the downstream intended signal – and potentially the ‘choice’ of the encryptome-stabilizing ligand. The specific composition of the GPCR encryptome therefore defines its subsequent interactibility with protein- and lipid kinases, phosphatases, ubiquitin ligases, regulators of small G proteins, and other novel effectors (Maudsley et al., 2005; Luttrell and Gesty-Palmer, 2010; Shenoy and Lefkowitz, 2011). The concept of signaling selectivity or ligand bias suggests that distinct ligands may alter the conformational equilibrium of the receptor in a manner distinct from the endogenous ligand, allowing the receptor to couple to a specific subset of its downstream effectors (Wei et al., 2003; Kenakin, 2007; Kenakin and Miller, 2010; Luttrell and Kenakin, 2011; Maudsley et al., 2012). This functional selectivity carries with it the promise of a revolutionary change for drug development: increasing the potential for new, more effective drugs, but also the knowledge that drug efficacy needs to be comprehensively characterized in order to avoid unintended side effects. In the context of the GPCR encryptome, ligand bias results as a function of the protein stoichiometric composition of this. Different protein components chaperoning and interacting with the core GPCR will thus determine the interaction/efficacy profile of the ligand attempting to interact and stabilize the particular encryptome.

While signaling bias using functionally selective xenobiotic ligands has been a topic of ongoing research for nearly two decades, it has become clear that natural ligand bias also occurs for GPCRs and likely represents another physiological level of receptor signaling complexity. In discussing the natural biased agonism of chemokine receptors (CCRs), Zidar et al. (2009) reinforce the posit that this selectivity is not merely a product of synthetic pharmacology (Kohout et al., 2004). In their research, Zidar et al. (2009) found that the endogenous chemokine ligands CCL19 and CCL21 for the CCR7 receptor, demonstrate a striking difference in activation of the GRK/β-arrestin2 system, despite of their similarity in promoting G protein stimulation and chemotaxis. Only CCL19 promotes desensitization of endogenous CCR7 in the human T cell lymphoma cell line H9 (Kohout et al., 2004), and causes fourfold more ERK1/2 phosphorylation than CCL21, through a β-arrrestin2 dependent mechanism (Kohout et al., 2004; Zidar et al., 2009; Zidar, 2011).

Endogenous ligand bias has also been shown for the Angiotensin II type 1 receptor (AT1R), which is physiologically implicated in the development of hypertension and the natural aging process (Mattson and Maudsley, 2009). Further research in AT1R, indicates that only a subset of AT1R signaling pathways are detrimental, thus by using biased ligands which inhibit these detrimental pathways, it may be possible to promote and enhance beneficial drug-based effects (Galandrin et al., 2016). Angiotensin peptide (1–7) [Ang(1–7)], which lacks the Angiotensin II (Ang II) critical C-terminal phenylalanine residue due to angiotensin-converting enzyme 2 (ACE2)-dependent cleavage is described to cause vasodilatory and cardioprotective effects (Santos et al., 2003, 2013). Ang(1–7) fails to promote G protein activation, behaving as a competitive antagonist for Ang II/Gα_i_ and Ang II/Gα_q_ pathways, it however selectively promotes β-arrestin activation (Galandrin et al., 2016). These researches explain that in nature, ligand bias has likely been exploited even before scientist have begun to uncover the clinical uses of ligand bias, underlining its importance.

### β-Arrestin Modulates Downstream Signaling of GPCRs Through Complex Assembly Regulation

**FIGURE 1:**
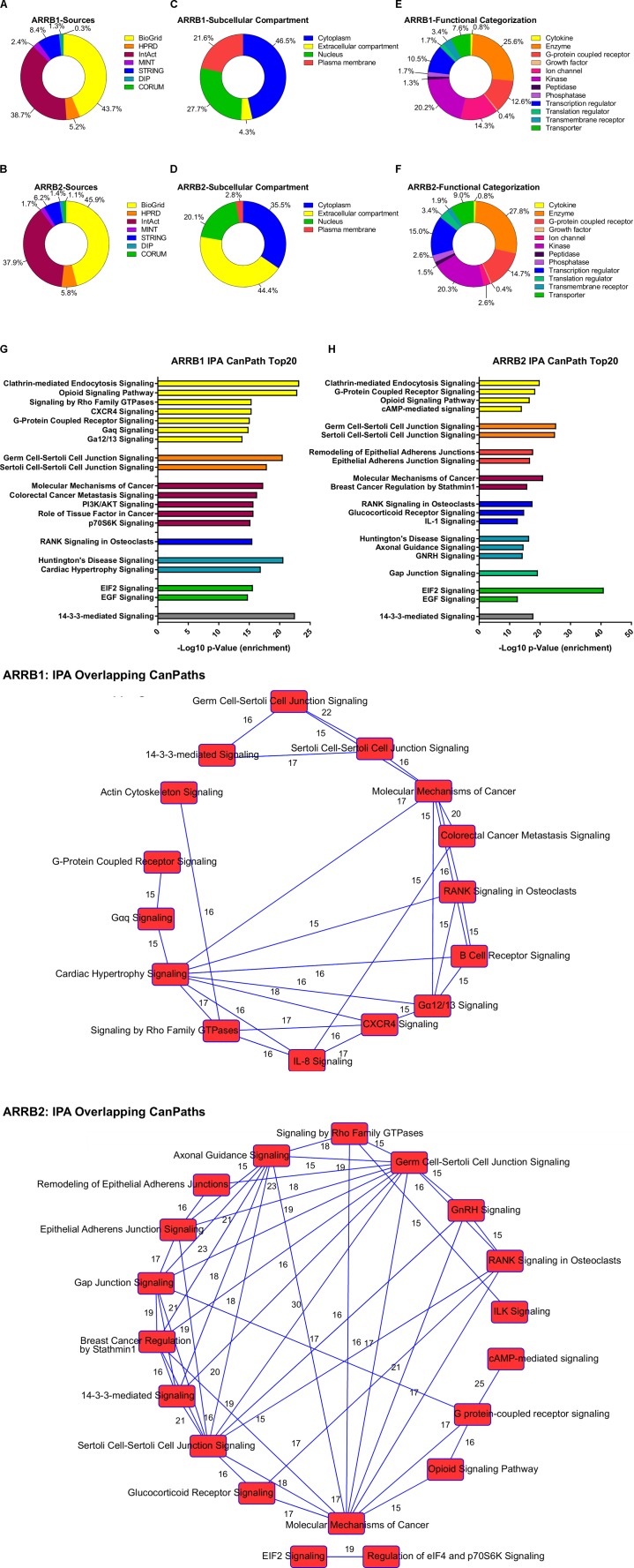
Ingenuity pathway analysis of β-arrestin1 and 2 interactome. The interactors of a protein are indicative of its function. Thus, to further investigate the function of β-arrestin1 we extracted these from seven sources: BioGrid (https://theBioGrid.org/), HPRD (Human Protein Reference Database: http://www.hprd.org/), IntAct (https://www.ebi.ac.uk/intact/), MINT (The Molecular INTeraction Database), STRING (https://string-db.org/), DIP (Database of Interacting Proteins: http://dip.mbi.ucla.edu/dip/), and CORUM (http://mips.helmholtz-muenchen.de/corum/). This resulted in a list of proteins which are proven interactors of β-arrestin1 (445) and 2 (625). **(A,B)** Shows the distribution of the aforementioned databanks for β-arrestin1 and 2 respectively. This dataset was further analyzed using Ingenuity Pathway Analysis (IPA). Where we extracted the following information: **(C,D)** the subcellular distribution – categorized in an unbiased manner using IPA protein annotation – of the interactors: Plasma Membrane, Nucleus, Cytoplasm, and Extracellular space, *i*, for β-arrestin1 and 2 respectively; **(E,F)** the functional annotation – again performed using unbiased IPA-based classification – of the interacting proteins: Cytokine, Enzyme, GPCR, growth factor, ion channel, kinase, peptidase, phosphatase, transcription regulator, translation regulator, transmembrane receptor, and transporter, β-arrestin1 and 2 respectively; and the Top 20 Canonical Pathways, β-arrestin1 and 2 respectively, related to this dataset organized in **(G,H)** a stacked bar chart, and **(I,J)** a network representation with a cut-off of 15 common genes between pathways, to increase the stringency, where the numbers depicted represent the amount of common proteins between the pathways.

**FIGURE 2:**
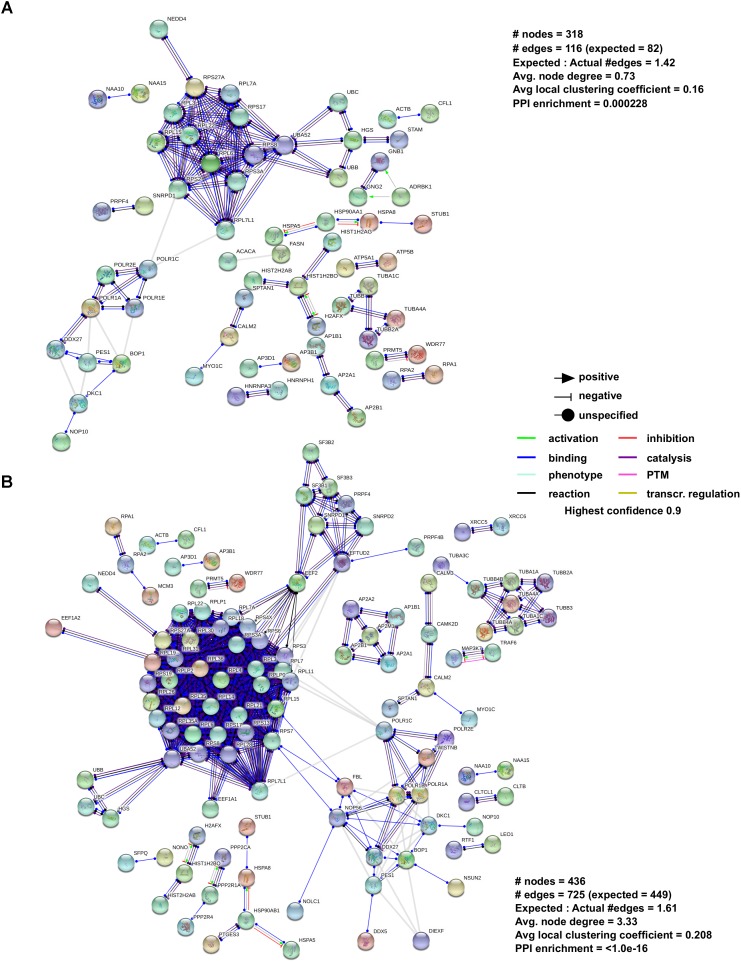
Analysis of β-arrestin1 and 2 interactome using STRING Interaction Network. To further analyze the networks of **(A)** β-arrestin1 and **(B)** β-arrestin2, STRING was used. To increase the stringency of the analysis the following settings were used: for Active Interaction Sources only “Experiments” and “Co-expression” were selected indicating we are only interested in data which is the result of experiments or are known to co-express, this to remove hypothetical data and suggestions. The minimum required interaction score was set to the highest level (0,9), and all unconnected nodes were removed from the network.

**FIGURE 3:**
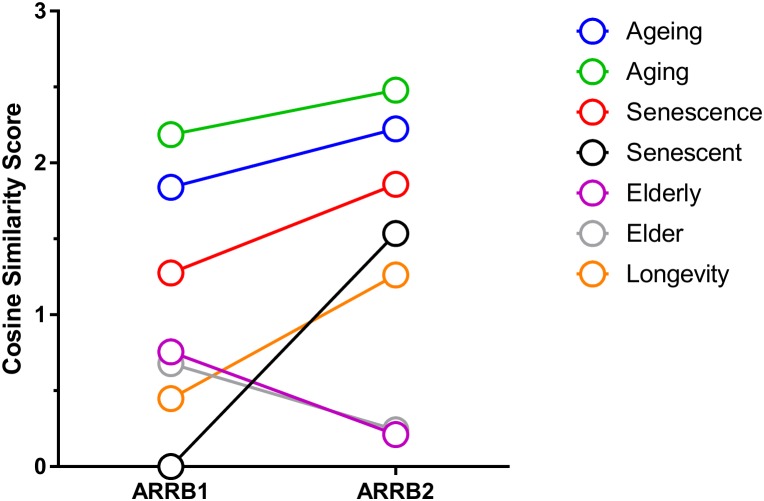
Role of β-arrestins in aging through GeneIndexer analysis of interactome metadata. Reanalysis of the obtained interactomes for β-arrestin1 (ARRB1) and β-arrestin2 (ARRB2), using aging-related interrogation terms with GeneIndexer, in a manner similar to that described previously (Chadwick et al., 2012). The following interrogation terms were used: Ageing, Aging, Senescence, Senescent, Elderly, Elder, and Longevity (i.e., long life). These terms (inclusive of most common spellings and synonyms) were used to obtain as much information as possible as human-generated text descriptions using natural language are often variant between research groups/authors. As seen in previous bioinformatics analyses these interrogation terms all give different results. In this figure, it becomes clear that β-arrestin2 has a stronger connection to aging compared to β-arrestin1, with the exception of the interrogation terms Elderly and Elder. For the generation of this figure, the cosine similarity between the top 10 proteins and every interrogation term was averaged in order to create this table. The cosine similarity scores linking the proteins to each word are listed in Supplementary Table S3.

As mentioned previously, β-arrestin can affect GPCR signaling in a manner independent of G protein-signaling that involves the physical scaffolding of multiple signal transduction proteins. Thus, it stands to reason that the signaling functionality of β-arrestin1 and 2 can be effectively interpreted via the analysis of the physical interactomes associated with these multidimensional transducers (Figures 1, 2). To assess the current state of the metadata concerning the known functional interactomes of the β-arrestin1 (Figure 1A) and β-arrestin2 (Figure 1B) we extracted binding partner identities from BioGrid^1^, HPRD (Human Protein Reference Database^2^), IntAct^3^, MINT (The Molecular INTeraction Database), STRING^4^, DIP (Database of Interacting Proteins^5^) and CORUM^6^. The cumulated known interaction partners for β-arrestin1 or β-arrestin2 are detailed in Supplementary Tables S1, S2, respectively. The diversity of subcellular distribution (Figure 1C – β-arrestin1; Figure 1D – β-arrestin2) and molecular function (Figure 1E – β-arrestin1; Figure 1F – β-arrestin2) of these interactors were categorized using Ingenuity Pathway Analysis (IPA)-based annotation. Perhaps the strongest divergence in cellular distribution of β-arrestin-specific binding partner between the two isoforms lies in the stronger plasma membrane representation of β-arrestin1 binding partners compared to β-arrestin2. In a similar regard, the greatest distinction of β-arrestin binding partner functionality resided in the much stronger ion channel function of β-arrestin1-binding partners compared to partners of β-arrestin2. To gain a signaling-based appreciation of the known β-arrestin-based interactomes, an IPA canonical signaling pathway investigation of the known binding partners (Figure 1G – β-arrestin1; Figure 1H – β-arrestin2) was carried out. Amongst the top 20 most strongly-populated signaling pathways it was evident that the β-arrestin1 binding cohort demonstrated a more profound propensity for G protein-associated functionality and cell cycle regulation compared to β-arrestin2. However, when the degree of potential signaling pathway overlap was assessed for the two curated β-arrestin interactomes (Figure 1I – β-arrestin1; Figure 1J – β-arrestin2) it was clear that a more interactive and closely associated functional network of activity was predicted for β-arrestin2. Such data suggests perhaps that β-arrestin2 may control a more coherent signaling response away from plasma membrane-associated receptors (see Figure 1D). This distinction in interactome coherence was also continued when the two β-arrestin interactome datasets were assessed using the STRING interaction platform (Figure 2A – β-arrestin1; Figure 2B – β-arrestin2). Hence, with respect to the network statistics of the two interactomes, the β-arrestin2-specific functional network, compared to the β-arrestin1 network, possessed a greater average node degree, a stronger local clustering coefficient and a more significant protein–protein interaction (PPI) enrichment probability. The natural language processing informatic platform GeneIndexer was employed to assess the relative strength of association between proteins β-arrestin1 or β-arrestin2 interactome datasets and gerontological biomedical concept terms. GeneIndexer^7^ enables the identification of biomedical text items, i.e., Gene Symbol identifiers of proteins, that possess latent semantic associations with the input interrogator concept term (i.e., Ageing, Aging, Senescence, Senescent, Elderly, Elder, Longevity). Latent semantic analysis is a natural language processing data extraction mechanism (Landauer et al., 2004) that exploits the concept of ‘Swanson Linking’ (Bekhuis, 2006) to identify cryptic connections between natural language concepts (i.e., Ageing, Aging *etc*….) and specific text entities, in this case Gene Symbol targets identified using the database of gene-word documents assembled in the GeneIndexer database (Cashion et al., 2013). GeneIndexer contains over 1.5 million Medline abstracts corresponding to over 21,000 mammalian genes (Cashion et al., 2013). GeneIndexer extracts both explicit and implicit gene-to-keyword (e.g., Aging) relationships from the literature using Latent Semantic Indexing (LSI: Homayouni et al., 2005). GeneIndexer scores individual proteins according to the strength of the association with the keyword query (e.g., Aging), whereby a Cosine Similarity (Landauer et al., 2004) score >0.2 typically indicates an explicit association (i.e., the word actually appears in the protein-related PubMed abstract), while a score between 0.1 and 0.2 typically indicates an implied relationship (Homayouni et al., 2005). Therefore using a basal Cosine Similarity cut-off of 0.1 we were able to assess the group (β-arrestin1 or 2 interactome) strength of correlation between the detailed aging-related interrogator terms (Figure 3) and the interactome datasets by summing the various individual protein Cosine Similarity scores extracted for each implicitly and explicitly-associated proteins from the interactome lists for each given gerontological concept input. One of the biggest challenges for the machine-based analysis of human-generated text concerns the vagaries of term use, e.g., authors may use divergent syntactic entities (words) to describe the same concepts (e.g., Aging or Ageing, Elderly or Elder). Hence to generate the most comprehensive LSI-based appreciation of gene-keyword associations we used a broad range of synonyms to extract the most amount of Gene Symbol data. Using this range of input terms (Aging, Ageing, Senescence, Senescent, Elderly, Elder, Longevity) we found that, using the cumulated Cosine Similarity scores for the interactome proteins associated with these input gerontological concept terms, for the majority of the terms (Aging, Ageing, Senescence, Senescent, and Longevity) a greater total association of the βarrestin2 interactome proteins with these concepts was observed compared to the βarrestin1 interactomes (Figure 3 and Supplementary Table S3). These unbiased results are effectively concordant with our previously derived STRING interactome analyses. This suggests, in line with recent reports (Luttrell et al., 2018b), that highly specific and contextual biological functionalities are likely attributable to either βarrestin1 or 2. Therefore, it seems that indeed a potential bias for β-arrestin2-dependent signaling may occur with respect to the specific context of aging. In the following sections we will discuss how, with specific reference to hypothesis-driven experimentation, the downstream signalers can be influenced by β-arrestin1 or β-arrestin2 interactome activity and subcellular distribution.

#### Non-receptor Tyrosine Kinase c-Src

As mentioned previously, β-arrestins also function as downstream signal transducers of GPCRs, allowing the formation of signal/function-specific encryptomes with a wide variety of signaling proteins, including c-Src family tyrosine kinases and components of the ERK1/2 and JNK3 (Jun N-terminal kinases 3) MAP kinase cascades (Luttrell et al., 1999; Luttrell and Lefkowitz, 2002). Through the recruitment of these kinases to GPCRs after agonist-binding, β-arrestins grant distinct signaling activities upon the receptor – discriminating them physically from G protein-dependent functional GPCR forms (Luttrell and Lefkowitz, 2002). This paradigm was illuminated by the initial observation that β-arrestin can directly bind to Src family kinases and recruit them to agonist-occupied GPCRs. Stimulation of β2 adrenergic receptors (β2AR) triggered the co-localization of the receptor with both endogenous β-arrestins and Src kinases in clathrin-coated pits (Luttrell et al., 1999). This reflected the assembly of the prototypic signal-encrypted receptor-protein complex. These GPCR-based encryptomes have also been seen in other research where β-arrestins recruited Src to the neurokinin-1 receptors in KNRK cells (DeFea et al., 2000) and Src family kinases Hck and Fgr to the C-X-C motif chemokine-1 receptor (CXCR1) in neutrophils (Barlic et al., 2000).

#### Mitogen Activated Protein (MAP) Kinases

The MAP kinases are a family of serine/threonine kinases involved in the transmission of external signals regulating cell division, growth, differentiation, and apoptosis. Mammalian cells contain three major categories of MAP kinases: ERKs, JNKs, and p38/HOG1 MAP kinases (Pearson et al., 2001). In 2000, it became clear that β-arrestins have the ability to function as scaffold proteins for some MAP kinase units. In KNRK cells the stimulation of proteinase-activated receptor 2 (PAR2) promotes encryptome formation containing the internalized receptor, β-arrestin1, Raf-1, and activated ERK1/2, which is required for ERK activation (DeFea et al., 2000). Similar results have been gained for the angiotensin II type 1A receptor (AT1aR) in HEK293 and COS-7 cells, where stimulation resulted in encryptome formation containing the receptor, β-arrestin2, and ERK cascade component kinases: cRaf-1, MEK1 (Mitogen Activated Kinase Kinase 1) and ERK2 (Luttrell et al., 2001). β-Arrestins can sequester ERK in the cytosol, by scaffolding Raf1, MKK1 and ERK (Luttrell et al., 2001). In this manner β-arrestin also scaffolds JNK1/2 through mitogen-activated protein kinase kinase (MKK) 4 and 7 (Kook et al., 2013). Furthermore, the activation of p38 signaling also appears to be dependent on β-arrestin (Sun et al., 2002; Bruchas et al., 2006).

#### E3 Ubiquitin Ligases

While ubiquitination was long thought to control only protein degradation, it has now become clear it also plays a role in productive signal transduction cascades. β-Arrestins can act as adapters for E3 ubiquitin ligases such as MDM2, which mediates ubiquitination of p53 and as such plays a role in DDR (Shenoy et al., 2001) by controlling p53 expression levels. The complexes that β-arrestin and these E3 ligases create, are essential for mediating ubiquitin-dependent signaling. For instance, β-arrestins are essential in the ubiquitination of receptors, where these scaffolds act on late endosomal population as a lysosomal degradation signal for the receptor. When focusing back on MDM2, ubiquitination of β-arrestin2 is also required for clathrin-mediated β2AR internalization (Shenoy et al., 2001). However, AIP4 E3 ligases are necessary for endosomal sorting of CXCR4, after which it can be sorted to lysosomes (Marchese et al., 2003). For this sorting, an interaction of β-arrestin1 with Signal transducing adapter molecule 1 (STAM1) is necessary, which is part of the endosomal sorting complex (Malik and Marchese, 2010). This connection between β-arrestin1 with CXCR4 also appears in our interactome metadata analysis of the β-arrestin1 interactome specifically (Figures 1G,J).

#### Nuclear Factors

β-arrestin2 has been shown to possess the ability to inhibit nuclear factor κB (NF-κB) signaling through the physical stabilization of IKBα (NFKB inhibitor alpha) (Gao et al., 2004). Next to β-arrestin2, its family co-member β-arrestin1 can directly influence epigenetic modifications through a nuclear interaction with histone acetylases and de-acetylases which influence chromatin structure (Kang et al., 2005). Moreover, β-arrestin1 is also necessary for endothelin-1-induced NF-κB activation in ovarian cancer cells since Cianfrocca et al. (2014) discovered a previously unrecognized pathway dependent on β-arrestin1 to sustain NF-κB signaling in ovarian cancer.

β-Arrestin can also control NF-κB-mediated signaling through AT1aR activation, which recruits this scaffold protein. This was analyzed in rat vascular smooth muscle cells. Genetic knockdown of β-arrestin1 and 2 inhibited Ang II-induced p64 NF-κB nuclear localization. The internalization of the activated AT1AR mediated by β-arrestin, stimulated the NF-κB signaling pathway, engendered a nuclear localization of the transcription factor and resulted in the initiation of COX-2 (Cytochrome c oxidase subunit 2) protein synthesis. As such, this research line linked receptor internalization with the NF-κB pathway (Morinelli et al., 2013).

In addition, β-arrestin2 exerts anti-inflammatory action through the prevention of NF-κB activation. This action is mediated through the direct inhibition of p38 MAPK. On the other hand, a simultaneous anti-inflammatory effect can also be initiated through cAMP and PKA activation via G protein signaling which exerts an inhibitory effect on NF-κB (Campo et al., 2015). Such output signaling complexity reinforces the need for a more in-depth understanding of the spatiotemporal dynamics of GPCR signaling before rational design of engineered efficacy drugs is feasible.

#### Receptors and G Protein Subunits

The epidermal growth factor receptor (EGFRs) can be functionally transactivated by GPCRs as well as being functionally regulated by β-arrestins, through the stimulation of a transmembrane matrix metalloprotease which causes cleaving of membrane bound EGF ligand (Noma et al., 2007). In addition to this, it has become clear that β-arrestin can mediate non-canonical G protein signaling, where they promote this novel form of cell signaling by both the Parathyroid hormone 1 (PTH1) (Wehbi et al., 2013) and V2 vasopressin receptor (Feinstein et al., 2013) from cytoplasmic endosome structures. This effect was lost after knockdown of β-arrestin (Feinstein et al., 2013).

The β2AR has been proposed to maintain an active conformation, even in endosomes distant from the plasma membrane, which has the ability to signal productively through G proteins to generate cAMP (Irannejad et al., 2013). This suggests that trafficking of the receptor to endosomes, via β-arrestins, facilitates the stabilization of a receptor that is not necessarily inactivated and ready to be recycled, but one that is still able to activate G protein signaling. This signaling paradigm appears to be facilitated by a Receptor – G protein – β-arrestin complex (Wehbi et al., 2013), thus completely countering the classic paradigm of β-arrestin as ‘arresting’ G protein signaling (mentioned in see section “Introduction”).

### β-Arrestin Dependent Signaling in Therapeutic Development

#### Type 1 Parathyroid Hormone Receptor

β-Arrestin dependent signaling-selective ligands have been investigating in the attempt to design precision *‘tailored efficacy’* therapies that possess a more focused efficacy with fewer off-target side effects. Perhaps one of the best characterized arrestin pathway-selective biased agonist is that mediated through the type 1 human parathyroid hormone receptor (PTH1R). The PTH1 receptor regulates calcium homeostasis and bone metabolism. Gesty-Palmer et al. (2006) reported that [D-Trp^12^,Tyr^34^]bovine PTH(7-34) showed β-arrestin bias, acting as an inverse agonist for PTH1R-Gα_s_ coupling (Appleton et al., 2013). Intermittent administration of bPTH(7-34) *in vivo* increased bone mass, osteoblast number, in the absence of osteoclast proliferation and bone resorption. These effects are in stark contrast to those engendered by similar treatment with endogenous human PTH(1-34) [hPTH(1-34)], which is the current conventional therapy for osteoporosis (Gesty-Palmer et al., 2009). Research has subsequently demonstrated that this diverse and effective functional activity in the PTH1R is mediated through the creation of a highly-translatable molecular signature of altered protein expression patterns that is dependent on a functional β-arrestin2 signaling capacity (Gesty-Palmer et al., 2013; Maudsley et al., 2015, 2016). This research has thus allowed GPCR-based drug design to enter the phase of multidimensional therapeutic targeting to engender highly selective long-lasting proteomics-based remediation of equally complex disease protein profiles.

#### μ-Opioid Receptor

The μ-opioid receptor (MOR) can be activated by both endogenous enkaphalin peptides and xenobiotic opiates such as morphine or fentanyl (Darcq and Kieffer, 2018). G protein agonism and receptor internalization have been demonstrated for enkephalins and the opiate etorphine, however a lack of receptor agonist-induced internalization was found for the opiate agonist morphine (Keith et al., 1996; Darcq and Kieffer, 2018). This could be explained by the poor ability of morphine to stimulate receptor phosphorylation and thus its recruitment of β-arrestin (Zhang et al., 1998), and is hence a good example of negative β-arrestin bias. This connection between β-arrestin and opioid signaling was also extracted from our obtained β-arrestin interactome dataset (Figures 1G–J). Despite this, mice lacking β-arrestin2 demonstrated a marked increase and prolongation of morphine-induced analgesia, indicating, notwithstanding its bias, that morphine is capable of stimulating β-arrestin-mediated desensitization (Bohn et al., 1999). This discovery indicates that ligands showing strong bias away from β-arrestin, toward G proteins, might offer enhanced analgesia, where it may be possible to separate the therapeutic efficacy from the negative side effects of such agents (Bruns et al., 2006; Schmid et al., 2017). One such compound has been discovered, named herkinorin, where it diverts signaling activities away from side effects, such as tolerance, respiratory suppression and constipation, which are mediated by β-arrestin2 (Bohn et al., 2000; Raehal et al., 2005; Groer et al., 2007; Manglik et al., 2016; Schmid et al., 2017). Furthermore, PZM21, a potent G_i_ activator showed minimal β-arrestin2 recruitment, causing analgesic effects in absence respiratory depression, and is as such another example of β-arrestin independent therapeutics (Manglik et al., 2016).

#### Angiotensin II Type 1 Receptor

The AT1R possesses a strong trophic role in the control of blood pressure and electrolyte homeostasis, and thus has been widely targeted in the strategic development of pharmacotherapeutic treatment for hypertension via the employment of classical ‘antagonists’ such as valsartan and losartan. Ang II typically signals through the AT1R via Gα_q_-mediated activation of phospholipase C (PLC) and induces receptor phosphorylation by GRKs, thus recruiting β-arrestin to the receptor. This β-arrestin recruitment desensitizes Gα_q_ signaling, causes receptor internalization and induces non-G protein-dependent β-arrestin signaling (Anborgh et al., 2000; Sakmar et al., 2002). Sar1, Ile4, Ile8-AngII (SII) was identified as a perfectly β-arrestin biased ligand (Holloway et al., 2002), stimulating β-arrestin signals such as ERK1/2 activation in the absence of G protein signaling (Wei et al., 2003; Ahn et al., 2004). Such discrete signaling has been elegantly demonstrated both *in vivo* and *in cellulo*. SII has been found to induce contractility via β-arrestin signaling in isolated cardiac myocytes, indicating a significant effect on cardiac function (Rajagopal et al., 2006). Several of the SII-activated signaling pathways appear to be pro-survival, cytoprotective, and/or proliferative, i.e., Src, ERK1/2, Akt, and phosphatidylinositol-3-OH kinase (PI3K) (Lefkowitz and Shenoy, 2005; Lefkowitz et al., 2006; DeWire et al., 2007), and are thus worthy of further investigation for drug-development.

Moreover, a potent, selective β-arrestin biased ligand of the AT1R has been recently discovered, i.e., TRV120027 (Sar-Arg-Val-Tyr-Ile-His-Pro-D-Ala-OH), which competitively antagonizes G protein signaling through the AT1R, yet stimulates β-arrestin recruitment, activating several kinase pathways in a β-arrestin-dependent manner. This biased ligand increased cardiomyocyte contractility *in vitro*. *In vivo* characterization of the ligand, in rats, showed a reduction in mean arterial pressure, and most importantly (and unlike other unbiased antagonists) TRV120027 increased cardiac performance and preserved cardiac stroke volume (Violin et al., 2010). Unfortunately, even though this drug showed great promise, TRV120027 failed in the clinical phase 2b. Such an initial setback is unlikely to diminish further interest in refining the efficacy profile of GPCR-targeting biased agents.

#### β-Adrenergic Receptor

Classical orthosteric βAR antagonists have long been employed as effective pharmacotherapeutics for numerous cardiovascular conditions including hypertension (Williams et al., 2004), angina (Gibbons et al., 2003), and heart failure (Bristow, 2000). They possess the ability to block the harmful G protein-mediated effects of superfluous catecholamine stimulation in the heart and other organs. The widely used βAR ‘blocker’ Carvedilol (Coreg^®^) has been demonstrated to possess unique survival benefits in congestive heart failure, through a β-arrestin-selective pathway in absence of the Gα_s_-dependent activation of adenylate cyclase (AC) (Wollert and Drexler, 2002; Wisler et al., 2007). This bias potentially explains its unique clinical effectiveness in heart failure and other cardiovascular disorders.

While for some cardiovascular diseases most so-called ‘β-blockers’ (βAR antagonists) are effective, in others only a subset of such agents exert favorable actions. The βAR receptor has also been targeted to treat asthma, a chronic inflammation of the airways. The use of glucocorticosteroids and β2AR agonists represent the cornerstones of asthma therapy (Thanawala et al., 2014). Recently however, research has been performed into the possibility of using biased ligands in the treatment of asthma. Studies have shown that nadolol and ICI-118,551 are inverse agonists at βAR for both G protein- and β-arrestin-dependent pathways. Interestingly, while carvedilol and propranolol share this inverse agonistic activity, they can still activate ERK1/2 through β-arrestin signaling (Galandrin and Bouvier, 2006; Wisler et al., 2007). Further investigation into murine models for asthma, showed that nadolol, but not propranolol, reduced airway hyperresponsiveness, a functional subset of the asthma symptomologies. This beneficial effect of nadolol in comparison to propranolol is likely related to the ability of the latter to activate ERK1/2 signaling. Thus, blocking β-arrestin-mediated signaling via βAR might be advantageous in asthma treatment (Walker et al., 2011; Thanawala et al., 2014).

## β-Arrestin in Aging

With advancing age, as well as age-related pathophysiology, an increased expression and functional impact has been shown for β-arrestin and several other GPCR scaffolding proteins (e.g., GRKs) (Schutzer et al., 2001; Bychkov et al., 2008; Tsutsui et al., 2008; Lu et al., 2017). This finding poses an interesting pathophysiological question related to the ‘complexity theory’ of aging. As we age there is proposed to be a global homeostatic loss of complexity (Lipsitz and Goldberger, 1992; Manor and Lipsitz, 2013; Sleimen-Malkoun et al., 2014), mediated by the entropic breakdown of metabolic energy generation and damage repair systems. However, simultaneous with this global reduction homeostatic stability, it is likely that multiple compensatory mechanisms, e.g., switching energy metabolism from the Type II Diabetes mellitus (T2DM)-affected glucometabolic system to less efficient systems such as fatty acid beta-oxidation or ketogenesis, are instigated to attenuate damage due to the loss of optimal signaling system efficiency. This aging-driven increase in metabolic molecular signaling diversity results in a counterintuitive rise in ‘allostatic’ complexity alongside the loss of global homeostatic complexity (McEwen, 1998; Karlamangla et al., 2002; Sturmberg et al., 2015; Shiels et al., 2017). Therefore, at a receptor-based microcosmic level, do the increasing levels of β-arrestin, with the resultant increase in signaling diversity, represent perhaps the ‘*tip of the spear’* of aging? Alterations in complexity and diversity of GPCR systems will likely impact nearly all physiological systems implicated in aging, thus an enhanced appreciation of how altered GPCR signaling diversity controls the aging process is vital for the future development of pharmacotherapeutics with augmented efficacy profiles in the specific contexts of stress and aging. As a caveat to this posit however, it is likely that alterations in GPCR β-arrestin bias during age-related pathology are not simply unilateral or unidirectional, as signaling diversity from GPCRs may be vital for *both* allostatic and homeostatic mechanisms. Thus, in addition to increased expression levels, decreases (reducing perhaps excessive allostatic load engendered by GPCR bias) in β-arrestin expression/functionality may also be important (Grange-Midroit et al., 2002). To dissect this complex temporal relationship between β-arrestin-mediated GPCR signaling diversity and the physiological aging context, it is first important to define the intersections between β-arrestin functionality and the major pathophysiological domains of the natural (as well as aberrant) aging process.

### β-Arrestin Intersection With the Hallmarks of Aging

#### Oxidative Stress

The elevated presence of ROS in nearly every cell in the body needs to be countered to relieve the well-documented phenomenon of age-related oxidative damage (Redman et al., 2018). When the body is unable to balance these ROS with antioxidants, oxidative stress and damage occurs, leading to the deleterious modification of proteins, lipids, and nucleic acids. β-Arrestin has been shown to regulate oxidative stress by controlling NADPH oxidase 4 (NOX4), a major source of ROS in the heart. It was demonstrated that β-arrestin knockdown decreases ROS and NOX4 expression by 50% (Philip et al., 2015). Accumulation of ROS after UV and hydrogen peroxide (H_2_O_2_) treatment leads to the activation of multiple stress kinase cascades, such as the apoptosis signal-regulating kinase 1 (ASK1) signaling pathways, and then induce cell apoptosis (Tobiume et al., 1997; Chang et al., 1998; Ichijo, 1999; Morita et al., 2001). Through ASK1, β-arrestins prevent down-stream signaling and thus inhibits H_2_O_2_-induced cell apoptosis (Zhang et al., 2009).

#### DNA Damage

One of the most scrutinized hallmarks of aging is the accumulation of DNA damage. p53 is a central player in DDRs, where this protein is upregulated and activated by genotoxic stress. In cases of cellular stress, p53 induces an active transcriptional response of effectors promoting apoptosis, cell cycle arrest, senescence, and DNA repair (Speidel, 2015). MDM2 is a ubiquitin E3 ligase that targets p53 for proteasomal degradation (Haupt et al., 1997; Kubbutat et al., 1997). Hara et al. (2011) demonstrated that β-arrestin1-mediated signaling downstream of the β2AR, could trigger DNA damage and suppress p53 expression, which adds up to the accumulation of DNA damage. This by facilitating Akt-mediated activation of MDM2 and promoting MDM2 association with, and degradation of, p53, through its role as a scaffolding protein. Further investigation into the link between β-arrestin1 and p53, demonstrated an interaction between these two proteins *in cellulo* and *in vivo* in the brain, where 50% of the binding between β-arrestin1 and p53 occurs in the nucleus (Hara et al., 2011).

#### Metabolic Dysfunction

With respect to age-related somatic metabolic dysfunction, perhaps the most prevalent are Metabolic Syndrome and generic insulin resistance leading to T2DM (Bonomini et al., 2015). In both of these dysfunctional conditions there is a disruption of the ability/efficiency of insulin to stimulate insulin receptor signaling and effectively mobilize and use glucose as a primary energy source for mitochondrial oxidative phosphorylation (Feng et al., 2011). β-Arrestin1, can scaffold and recruit PI3K to the insulin growth factor-1 receptor (IGF-1R) in an agonist-dependent manner, thus facilitating the activation of PI3K and Akt. By doing so, β-arrestin1 is capable of weakening insulin-induced degradation of insulin receptor substrate-1 (IRS1) and thus promote downstream insulin signaling. Furthermore, β-arrestin1 binds to MDM2, as mentioned in Section “DNA Damage,” and competitively inhibits MDM2-mediated IRS1 degradation, and in doing so improves insulin sensitivity (Usui et al., 2004; Lefkowitz et al., 2006).

β-Arrestin1 has also been implicated in insulin secretion through the Glucagon-like peptide-1 receptor (GLP-1R), which has been the focus for both new anti-diabetic and anti-neurodegenerative therapies (Janssens et al., 2014). Sonoda et al. (2008) reported that β-arrestin1 mediated the ability of GLP-1 to stimulate cAMP and insulin secretion in pancreatic β cells. Moreover, a direct physical interaction between the receptor and β-arrestin was found in cultured INS-1 pancreatic β cells (Dalle et al., 2011). Quoyer et al. (2010) discovered that in addition to this, β-arrestin1 can mediated GLP-1 anti-apoptotic effects by phosphorylation of the pro-apoptotic protein Bad through ERK1/2 activation (Quoyer et al., 2010). Broca et al. (2009) reported that β-arrestin1 could control potentiation of glucose-induced long-lasting ERK1/2 activation controlling IRS2 expression, through the actions of pituitary adenylate cyclase-activating polypeptide (PACAP) (Broca et al., 2009). This year, Jones et al. (2018) indicated that compounds retaining GLP-1R at the plasma membrane through a reduction in β-arrestin recruitment were able to engender greater long-term insulin release patterns. These compounds elicited glycemic benefits in mice without related increases in signs of nausea which often occurs with GLP-1 therapies (Jones et al., 2018).

In addition to alterations of metabolism mediated by glycoregulatory disruption, global metabolic functions in aging are affected by changes in thyroid hormone activity. Thyroid hormones serve to control the generic metabolic rate in the body through the control of glucose/lipid/protein catabolism. With advancing age however there is an increased prevalence of hypothyroidism, which is furthermore associated with coronary heart disease, heart failure and cardiovascular mortality (Gesing et al., 2012). An animal model for hypothyroidism showed unaltered β-arrestin1 expression in the heart, yet a decrease was seen in the lung and an increase in the liver, this in contrast to the expression of GRK5 which shows significant up-regulation in lung and heart and a decrease in the liver shortly after birth. This confirms an independent regulation of the expression of these proteins and thus suggesting the presence of significant ‘active’ compensatory mechanisms (Penela et al., 2001). The potential functional correlation of these changes in β-arrestin1 are highly nuanced, given the fact that this scaffolding protein acts downstream of GRK’s in GPCR regulation (Michel-Reher et al., 1993).

#### Chronic Inflammation

Long-term uncontrolled inflammatory activity is a key trophic facet in the aging process. A chronic pervasive form of inflammation is so common among pathological aging phenotypes that the generation of the novel *portmanteau* description of this process as ‘inflammaging’ was warranted. β-Arrestins have been shown to act as scaffold proteins or signal transducers for key inflammatory signaling molecules in receptor tyrosine kinases (RTKs) signal transduction pathways, such as the NF-κB pathway (Gao et al., 2004). NF-κB is a ubiquitously expressed transcription factor that regulated genes involved in inflammation, immunity, and stress (Shoelson et al., 2003). The inflammatory NF-κB system has also been recently implicated in the basic metabolic control of the natural aging mechanism itself (Zhang et al., 2013). Campo and co-workers recently demonstrated that the anti-inflammatory action of β-arrestin2 appears to be mediated partially through the direct inhibition of p38 MAPK which prevents the activation of NF-κB, and partially through cAMP and PKA activation through G protein signaling, which also exerts an inhibitory effect on NF-κB (Campo et al., 2015).

Wang et al. (2015) reported that fenoterol, a βAR agonist used for relieving sudden asthma attacks, by stimulating βARs to relax bronchial smooth muscle, has the ability to inhibit 5-amino-1-b-D-ribofuranosyl-imidazole-4-carboxamide (AICAR)-induced AMPK activation and inflammatory cytokine production in cells. The AMPK pathway is involved in regulating inflammation in several cells lines (Wang et al., 2015). This inhibition, as well as the attenuation of tumor necrosis factor α (TNF)-α release, was abolished with the knockdown of β-arrestin2, indicating that β-arrestin2 likely facilitates the anti-inflammatory effects of fenoterol in AICAR-treated cells (Wang et al., 2016). Du et al. (2014) investigated the pro-inflammatory activity of fluoxetine (Prozac^®^), where they found that this antidepressant increased β-arrestin2 expression and enhanced the association of β-arrestin2 with transforming growth factor beta-activated kinase 1 (TAK1) binding protein 1 (TAB1) and disrupted the TAK1-TAB1 interaction, which attenuates the IκB (inhibitor of κB) degradation and NF-κB nuclear translocation (Du et al., 2014).

Further research in the role of β-arrestins in inflammation, has indicated that the anti- or pro-inflammatory dimensions of β-arrestin2 activity could be dictated by its ubiquitination status, which is linked to its ability to scaffold and localize activated ERK1/2 to receptorsomes (Jean-Charles et al., 2016). This was hypothesized since β-arrestin2 affects tumor necrosis factor receptor-associated factor 6 (TRAF6) in an anti-inflammatory manner, while physiologic β-arrestin2 promotes inflammation in disorders such as atherosclerosis and neointimal hyperplasia. In this specific context, the constitutive ubiquitination of β-arrestin2 furthermore augmented NFκB activation (Jean-Charles et al., 2016).

### β-Arrestin in Age-Related Disorders

#### Type II Diabetes Mellitus (T2DM)

The occurrence of T2DM increases with age, where the body builds up an insulin resistance (linked to insulin receptor desensitization mechanisms), as well as the presence of a secretory dysfunction of the excess plasma-borne glucose (i.e., microvascular disease) (Matthaei et al., 2000; Taniguchi et al., 2006). Recent studies have shown that the orphan receptor GPR40 might be an attractive target to enhance insulin secretion in T2DM patients. Research into the relationship of β-arrestin and this receptor have shown that β-arrestin2, together with GRK2, play an essential role in the regulation of agonist-mediated internalization, but not of the constitutive GPR40 internalization (Qian et al., 2014). This suggests that perhaps targeting and modulating this GPR40 through β-arrestin2, thus influencing the internalization, might be an interesting road to take, in treating T2DM.

Additionally, there is a crosstalk between the insulin/Insulin-like growth factor 1 receptors and signaling pathways including G proteins and β-arrestin (Blair and Marshall, 1997; Lin et al., 1998; Povsic et al., 2003; Ryall and Lynch, 2008). In diabetic mice, β-arrestin2 expression is severely declined, moreover knockdown of β-arrestin2 worsened insulin resistance, while administration of β-arrestin2 could rescue this phenotype and restore insulin sensitivity in the mice. Lastly, the formation of a new β-arrestin2 signal complex occurs after insulin stimulation. In this complex, β-arrestin2 scaffolds Akt and Src to the insulin receptor directly. Loss or dysfunction of β-arrestin2 disrupted the formation of this novel signal complex and caused a disturbance of insulin signaling *in vivo*, as such aiding in the development of insulin resistance and thus the progression of T2DM (Luan et al., 2009). Also, β-arrestin2 plays a central role in irisin-induced glucose metabolism in T2DM by regulating the p38 MAPK signaling (mentioned in see section “E3 Ubiquitin Ligases”), which according to Pang et al. (2017) might present a novel therapeutic target for diabetes.

Lastly, as mentioned in Section “Metabolic Dysfunction,” biased GLP-1R targeted ligands, diverting the signaling away from β-arrestin signaling, and allowing the GPL-1R to be retained at the plasma membrane, have shown promise for the treatment of metabolic dysfunction (Sonoda et al., 2008; Al-Sabah et al., 2014). This was further investigated by Zhang et al. (2015) using GLP-1R G protein-biased agonist called P5. In this research, it was shown that this biased ligand promoted G protein signaling comparable to GLP-1 and Exendin-4, yet demonstrated a significantly reduced β-arrestin response. This bias away from β-arrestin dependent signaling appears to be more effective than Exendin-4 at correcting hyperglycemia and lowering hemoglobin A_1c_ levels in diabetic mice after chronic P5 treatment (Zhang et al., 2015).

#### Age-Related Neurodegeneration

β-Arrestin2 has been shown to play a crucial role in the regulation of neurotransmitter receptors in the brain that are associated with the generation of age-related neurodegenerative phenotypes (Grange-Midroit et al., 2002). With advancing age, diverse and nuanced changes in β-arrestin expression have been noted. For example, a decrease in β-arrestin2 density has been found, which also occurs for most brain neurotransmitter receptors and specific G coupling proteins, indicating that this decline in expression of both receptor and receptor regulatory proteins is a common consequence of the senescent brain (Sastre et al., 2001). Alzheimer’s disease is a neurodegenerative debilitating disorder, which has been investigated thoroughly – unfortunately however no preventative therapy has been discovered yet despite this research. All efforts of helping AD patients are based on symptomatic treatments, in an attempt to slow down the progression of the disorder. Unfortunately, as AD is a neurodegenerative disorder, once the patients display symptoms, the disease has likely already progressed too far to be therapeutically reversed. Proteins being targeted for drug development are amyloid β, γ-secretase and the amyloid β precursor protein (APP). The aberrant processing of these proteins has been identified as a hallmark of AD development (Martin et al., 2013; Thathiah et al., 2013; Zhang et al., 2017).

Nervous systems functions, such as learning and memory, controlled by βAR are recently hypothesized to be mediated by G protein-independent signaling pathways. Liu et al. (2015) reported that memory retrieval induced the activation of β-arrestin signaling through the β1AR. β-Arrestin allows the stimulation of ERK signaling and protein synthesis, which, in this situation, leads to post-memory retrieval restabilization (Liu et al., 2015). In addition to this, β-arrestin2 knockout mice exhibit impaired memory retrieval in novel object recognition tests and in Morris Water Maze analyses. This reveals the potential therapeutic value of β-arrestin-biased ligands in the treatment of memory-related disorders (Liu et al., 2015). Further investigation into this relationship between β-arrestin and neurodegenerative disorders, has shown that β-arrestin2 expression is elevated in two independent cohorts of AD patients (Thathiah et al., 2013), and genetic variation of β-arrestin2 is associated with late-onset AD (Jiang et al., 2014).

When analyzing the relationship between β-arrestin and the AD biomarker protein amyloid β, β-arrestin2 overexpression leads to an increase in amyloid β peptide generation, the opposite was true for genetically silencing β-arrestin2 *in vitro* and *in vivo* in β-arrestin2 knockout mice (Thathiah et al., 2013). Moreover, β-arrestin2 has been shown to interact with GPR3 and β2AR GPCRs, which have both been implicated in AD pathology and mediate their effects on amyloid β generation. β-Arrestin2 also interacts with the anterior pharynx defective 1 homolog A (APH1A) subunit, part of the γ-secretase complex, which mediates the final catalytic step that liberates amyloid β from its precursor protein APP, and as a such, lies central to many amyloid β therapeutic strategies (De Strooper, 2003; Hur et al., 2012; Thathiah et al., 2013). Further investigating this interaction between β-arrestin2 and APH1A, the tri-cyclic antidepressant amitriptyline (Elavil^®^) has previously shown to cause strongly neurotrophic pharmacological activities, and is thus a possible treatment of neurodegenerative disorders such as AD. Acute cellular treatment of human neurons showed alteration in the physical interaction between the neurotrophic tyrosine kinase receptor 2 (NTRK2), and APP, and APH1A and β-arrestin2, suggesting that changes in this multi-protein complex may be associated with the beneficial therapeutic actions of amitriptyline (Martin et al., 2013).

Zhang et al. (2017) investigated the association of APP with GPCRs – they found that APP interacted with α_2A_AR. The association between these two proteins is promoted by agonist stimulation, but competes with β-arrestin2 binding to the receptor and thus negatively affects receptor arrestin-dependent internalization and desensitization (Zhang et al., 2017). The α_2A_AR plays an important role in controlling norepinephrine release and response. This discovery that APP can affect α_2A_AR internalization may have an impact on modulation of noradrenergic activity and sympathetic tone, but also affecting pain perception and decreasing epileptogenesis and anxiety (Hein, 2006; Masse et al., 2006; Gyires et al., 2009; Zhang et al., 2017). The noradrenergic system regulates arousal, learning and memory, which has also been implicated in regulating neuroinflammation (Ardestani et al., 2017). This loss of the noradrenergic tone may underlie AD progression at many levels (Ardestani et al., 2017; Zhang et al., 2017), and has further also been investigated by Ardestani et al. (2017) in the 5XFAD mouse model of AD. This research group used a partial agonist of the β1AR, Xamoterol, which restores the behavioral deficits of AD mouse models (Ardestani et al., 2017). Xameterol has been demonstrated to show bias away from the β-arrestin toward the cAMP pathway. Moreover, Ardestani et al. (2017) looked for a potential role of the partial agonist to counter neuroinflammation, where chronic administration reduced mRNA expression of neuroinflammatory markers, but also amyloid beta and tau pathology, which are used as markers for AD development, measured by regional immunohistochemistry (Ardestani et al., 2017). This G protein-dependence was further investigated by Yi and co-workers (Yi et al., 2017) – their work lead to the development of a brain-permeable G protein-biased β1AR ligand for the treatment of neurocognitive disorders (Yi et al., 2017). This receptor is fundamentally involved in the pathological features associated with AD, such as the cognitive deficits, and regulation of neuroinflammatory processes. These data therefore indicate that β1AR may be a promising therapeutic target for AD, where its activation may produce neuroprotective effects in neuroinflammatory disorders. In their recent paper, Yi et al. (2017) identified a possible functionally selective partial agonist for this receptor, namely the molecule STD-101-D1, which shows high brain penetration and inhibits TNFα. However, further research needs to be performed in order to confirm its therapeutic potential (Yi et al., 2017).

The cognitive deficit in AD is thought to be caused by the degeneration of the cholinergic receptor system. This pathology is thought to be linked to a loss of signaling through the cholinergic M_1_-muscarinic receptor subtype. Current studies offer an alternative mechanism involving the M_3_-muscarinic receptor subtype that is expressed in numerous brain regions including the hippocampus. The M_3_-muscarinic receptor knockout mouse demonstrates a deficit in fear conditioning learning and memory, which appears to be dependent on receptor phosphorylation/arrestin signaling. This opens the potential for biased M_3_-muscarinic receptor ligands that direct phosphorylation/arrestin-dependent (non-G protein-dependent) signaling as being beneficial in cognitive disorders (Poulin et al., 2010).

#### Osteoporosis

Human PTH regulates calcium homeostasis as well as bone formation and resorption via activation of the PTH1R – as such it is a possible target in treating the highly prevalent age-related bone disorder osteoporosis (Gesty-Palmer et al., 2005). Functions of arrestin in bone were first described in UMR 106-H5, an osteoblastic cell line, where β-arrestin2 was found to be involved in PTH1R desensitization (Bliziotes et al., 1996). Activated PTH1Rs recruit both β-arrestin1 and 2, which leads to the clathrin-dependent internalization of this complex, and arrestin-dependent scaffolding of the ERK1/2 cascade (Ferrari et al., 1999; Vilardaga et al., 2002; Gesty-Palmer et al., 2006). This ERK1/2 activation occurs via multiple independent pathways, involving PKA, PKC and/or arrestins (Verheijen and Defize, 1997; Lederer et al., 2000; Gesty-Palmer et al., 2009). Thus, as previously mentioned, β-arrestins have the ability to serve as multifunctional scaffolding proteins, in addition to their desensitizing actions. In this context, β-arrestins link the PTH1R to signaling molecules independently of classic G protein-mediated second messenger-dependent pathways (Bohinc and Gesty-Palmer, 2013). This ability to dissociate arrestin- and G protein-dependent PTH1R signaling in bone will likely influence future therapeutic design for osteoporosis treatment.

We have previously mentioned the role for β-arrestin in PTH signaling and in osteoporosis treatment (Gesty-Palmer et al., 2006; Gesty-Palmer et al., 2009; Luttrell and Gesty-Palmer, 2010). In addition to this, when PTH was administered intermittently to β-arrestin knockout mice the anabolic effects on the trabecular bone compartment were blunted, indicating that the recruitment of arrestins may be required to maintain a positive bone remodeling balance (Ferrari et al., 2005; Kostenuik et al., 2007). Unlike PTH, a β-arrestin biased PTH isoform (bPTH7-34) appears to uncouple the beneficial anabolic effects of the PTH1R activation from its catabolic and calcitropic negative side effects, thus confirming an effective role for β-arrestin in osteoporosis treatment (Gesty-Palmer and Luttrell, 2011; Luttrell et al., 2018a). When we analyze the interacting proteins of β-arrestin1 and 2, this clinically-relevant connection to osteoporosis reappears using the unbiased analysis of interactomic metadata (Figures 1G–J).

#### Cardiovascular Disorders

While both β-arrestin1 and 2 are expressed throughout the cardiovascular system, increases in β-arrestin2 expression have been found in aged aortas (Gao et al., 2004; Aplin et al., 2007). Additional research has indicated that this expression alteration also occurs within aged hearts (Dobson et al., 2003). Several GPCRs regulated by these two β-arrestins play immensely important roles in cardiovascular physiology and homeostasis. Contractility or cardiac function is tightly controlled by β1- and β2AR at the plasma membrane of cardiac myocytes. Cardiac structure and morphology on the other hand are regulated by AT1Rs in the cardiac fibroblast and endothelial cell membranes (Lymperopoulos and Bathgate, 2013). *Ex vivo* studies revealed that SII (mentioned in see section “Angiotensin II Type 1 Receptor”) can stimulate the contractility of isolated cardiac myocytes via the AT1R and β-arrestin2 (Rajagopal et al., 2006) and MAPK activation in perfused hearts (Aplin et al., 2007). This data indicates that β-arrestin bias may have a substantial impact in a physiological cardiovascular setting.

Dobson et al. (2003) also observed a concomitant age-dependent decrease in β1AR and adenylyl cyclase mRNAs. Taking these results together, this strongly suggests that these expressional changes in β1AR, adenylyl cyclase and β-arrestin play a causal role in the declined adrenergic signaling seen in aged hearts (Dobson et al., 2003). Moreover, in the context of myocardial infarction in a mouse model of heart failure, the molecular deletion of β-arrestin1 at the genetic level leads to increased survival as well as decreased cardiac infarct size, myocardia apoptosis, and adverse cardiac remodeling (Bathgate-Siryk et al., 2014). β-Arrestin2, however, shows the opposite, where it mediated EGFR transactivation by β1AR and thus plays a protective role against cardiac apoptosis (Noma et al., 2007). Carvedilol preferentially stimulates βAR through β-arrestin. But even more than that, it induces the transition of the β1AR from a classical Gα_s_-coupled receptor to a Gα_i_-coupled receptor, thus stabilizing the distinct β-arrestin-dependent receptor conformation. This Gα_i_ recruitment has not been shown for any other screened βAR ligand so far, nor is it required for β-arrestin activation by the β2AR (Wang et al., 2017). This advocates that the concept of β-arrestin-bias may need to be refined to include the selective bias of receptors toward distinct G protein subtypes.

Additionally, the α1ARs in the cardiovascular system function as stimulatory receptors and regulate vascular smooth muscle contraction. As such they aid in the elevation of systemic blood pressure through coupling to the Gα_q/11_ protein – PLC – Ca^2+^ mobilization pathway (Piascik and Perez, 2001). Antagonists of this receptor type, and thus biased ligands, have the ability to lower blood pressure in hypertension, hence these ligands biasing toward G protein can be used to treat hypotension by causing vasoconstriction (Jensen et al., 2011).

β-Arrestins also possess the ability to regulate oxidative stress, one of the prime driving factors in metabolic aging, in a NOX4-dependent manner, instigating an increase fibrosis in heart failure. This was further investigated in cardiac fibroblasts, where β-arrestin overexpression increased mitochondrial superoxide production twofold, which stimulates collagen deposition, thus leading to myocardial fibrosis, a precursor to heart failure (Ichijo, 1999; Philip et al., 2015). The recruitment of β-arrestin to the AT1R possibly engages a pathway where Src phosphorylates and activates Akt, which in turn phosphorylates endothelial nitric oxide synthase (eNOS) (Haynes et al., 2003; Suzuki et al., 2006), which could further provide a connection between AT1R-arrestin function and cardiovascular tone via nitric oxide regulation (Violin et al., 2010). Consistent with β-arrestin bias of TRV120027, eNOS activation by this ligand is eliminated by β-arrestin2 silencing. However, Ang II-stimulated eNOS phosphorylation is reduced by approximately 50% after β-arrestin2 knockdown, suggesting that the Ang II receptor can activate eNOS by both β-arrestin2-dependent and –independent pathways (Violin et al., 2010).

The endocrine peptide apelin has received considerable recent pharmacotherapeutic attention due to its potential therapeutic abilities for disorders such as pulmonary arterial hypertension and heart failure (Yang et al., 2015). Infusion of apelin leads to vasodilation, as well as cardiac inotropy without hypertrophy (Tatemoto et al., 2001; Szokodi et al., 2002; Berry et al., 2004; Ashley et al., 2005; Jia et al., 2006; Atluri et al., 2007; Maguire et al., 2009; Japp et al., 2010; Perjes et al., 2014; Brame et al., 2015). The predominant isoform in the human cardiovascular system is [Pyr^1^] apelin-13 (Maguire et al., 2009). Upon activation with its endogenous ligand, the apelin receptor is rapidly internalized through β-arrestin (Zhou et al., 2003; Lee et al., 2010). This receptor desensitization may therefore limit clinical efficacy of apelin-based agents. By creating a G protein-biased small molecule apelin agonist, Read et al. (2016) hypothesized that they could provide a solution to this limitation. Such apelin receptor modulators were shown to have beneficial cardiovascular action compared to the native peptide in humans *in vivo* (Ceraudo et al., 2014; Chen et al., 2014). One such biased small molecule, CMF-019, has therefore been proposed as an effective future biased receptor therapeutic (Read et al., 2016).

#### Cancer

Next we will shortly discuss the role for β-arrestins in the development of cancer. As a downstream signaler of GPCRs and activator of tyrosine kinase Src it stands to reason that these scaffolding proteins might play a role in cancer development. The activation of the GPCR endothelin-A receptor by endothelin 1 appears to play an important role in ovarian tumorigeneses and advancement, through the recruitment of β-arrestin. The role of this scaffold protein lies in the formation of trimeric complexes through one of which is through Src-interaction leading to transactivation of EGFR and β-catenin tyrosine phosphorylation (Rosano et al., 2009). Additionally, β-arrestin can contribute to β-catenin stabilization, which is part of the cell invasion program, through a physical association with axin. Silencing of β-arrestin1 and 2 completely nullifies the involvement of β-arrestin in the relationship between endothelin-A receptor and β-catenin pathway in this invasive program. One antagonist, ZD4054, allows this abrogation to take place and as such may open up new therapeutic opportunities for the treatment of ovarian cancer (Rosano et al., 2009).

The relationship between EGFR and β-arrestin1 has further been analyzed by Buchanan et al. (2006) in relation to colorectal carcinoma. The prostaglandin E2-induced transactivation of EGFR in these cancer cells are facilitated through a c-Src-dependent mechanism, which regulates cell migration and differentiation, both of which are important in tumor development. In their research, Buchanan et al. (2006) found that the prostaglandin E/β-arrestin1/c-Src signaling complex formation is crucial in this transactivation of EGFR and thus implicates a functional role for β-arrestin1 as a moderator of cell migration and metastasis.

Furthermore, the involvement of β-arrestin signaling in IGF-1R downstream signaling in Ewing’s sarcoma implicates that we should focus on a ligand showing bias away from β-arrestin. This was hypothesized as the association of the IGF-1R and β-arrestin1 due to the anti-IGF-1R antibody figitumumab, which has the following negative results: (i) receptor ubiquitination and degradation, and (ii) a decrease in cell viability and ERK signaling activation through β-arrestin1. As such, this signaling bias, i.e., β-arrestin1 regulation, suggests a possible therapeutic strategy to enhance response to anti-IGF-1R therapies (Zheng et al., 2012).

In addition to the demonstration that the EGFR receptor forms functional complexes with GPCR systems (Della Rocca et al., 1999; Prenzel et al., 1999; Maudsley et al., 2000; Roudabush et al., 2000), further GPCR-RTK associations, e.g., with platelet-derived growth factor receptors (PDGFRs) were subsequently shown to mediate a strong linkage between these signaling domains (Conway et al., 1999; Maudsley et al., 2000; Waters et al., 2003). PDGFR signaling has been strongly linked with gastric cancer (Qian et al., 2018), gliomas (Heldin et al., 2018), soft-tissue sarcomas (Vincenzi et al., 2017) and colorectal cancer (Manzat Saplacan et al., 2017). As β-arrestins form a crucial part of GPCR-based signaling it is not surprising that they possess the ability to modulate PDGFR signaling in concert with c-Src (Waters et al., 2005; Pyne and Pyne, 2017).

## Discussion

G protein coupled receptors are the most common molecular targets of clinically-relevant drug therapies – at this moment nearly 50% of all therapies are directed at the heterotrimeric G protein signaling GPCR states (Ayoub, 2018). However, it is now clear that the seven-transmembrane receptors can signal through β-arrestins in a clinically-relevant manner. By targeting GPCRs through their β-arrestin signaling, the number of drugs can perhaps be doubled. In addition to increasing the number of drugs, the specificity of these drug can also increase, thus aiding in the development of true ‘precision medicine,’ a medical model that proposes the customization of therapeutics and healthcare, with medical treatments being adapted to the individual patient or smaller patient groups clustered by similar molecular signatures. It has also become clear that *‘biased ligands’* can selectively activate G protein and/or β-arrestin functions, thus eliciting novel biological effects for even the best studied GPCRs (Violin and Lefkowitz, 2007). In this review, we have focused on specific β-arrestin functions, more specifically its important role in aging, and thus the possibility in treating age-related disorders, such as neurodegeneration, and cardiovascular disorders.

It has become clear that depending on the goal of the therapy, it is important to push the *‘bias’* toward β-arrestin, as is the case for osteoporosis discussed in Sections “Type 1 Parathyroid Hormone Receptor” and “Osteoporosis,” or away from β-arrestin, as is the case for the μ-opioid receptor (see section “μ-Opioid Receptor”). While they have only been recently identified, biased ligands have actually been in pharmaceutical drug development for a long time, but they were labeled as classical ‘antagonists’ (Andresen and Luttrell, 2011). The most important question now for biased ligand design is ‘how much bias is necessary for a successful therapy?’ While 100% of β-arrestin bias will most likely cause adverse effects, 60% bias might be ideal for a specific therapy. Furthermore, should drugs be designed to incorporate, or avoid, activation of the β-arrestin signaling pathways? We believe that completely removing one of the two signaling pathways, could be more harmful than advantageous (Maudsley et al., 2012). The body has been designed to function through both pathways, thus completely removing one or the other, could be catastrophic.

G protein coupled receptors located at the plasma membrane function as information channels running between the external environment and the interior of the cell. This signal transduction is based on the physical interaction of receptors with the intracellular effectors after activation by the productive interaction with the extracellular ligands. Additionally, it has become clear that membrane receptors can assume multiple conformations, which are each possibly capable of interacting with a specific subset of possible effectors (Luttrell and Kenakin, 2011). Thus, it is important to note that GPCRs interact with a broad range of diverse proteins other than heterotrimeric G proteins or β-arrestins. It thus stands to reason that there may be other effectors which could elicit specific signaling effects as well. These factors may likely be scaffolding proteins that may interact with GPCRs in diverse physiological contexts (e.g., oxidatively stressed), as opposed to the general ‘background’ receptor interactivity of G proteins or β-arrestins. As such, ligand efficacy has truly become pluridimensional, which evokes more possibilities for nuanced therapeutic design, but also increases the complexity of ligand classification and design.

While ligand bias is now being widely explored and the development of biased therapeutics has begun, since the discovery of β-arrestin, no concerted systematic research has been performed with respect to the possibility that there might be more therapeutically-valuable scaffolding proteins which interact with GPCRs. These scaffolders might thus be potent downstream effectors and cause signaling cascades of their own just like β-arrestin, i.e., sodium-hydrogen exchange regulatory factor 1 (NHERF1) (Maudsley et al., 2000), Multi-PDZ domain protein 1 (MUPP1) and GRK interacting transcript 2 (GIT2) (van Gastel et al., 2018). The identification of these proteins might cause an even larger increase in the discovery of more effective and selective therapeutics. This allows us to believe that we are on the verge of a new age of GPCR targeted pharmaceuticals that will exploit the ligand bias to adapt drug efficacy by enhancing therapeutically beneficial signals and blocking harmful ones (Rajagopal et al., 2010).

## Author Contributions

JvG, JOH, HL, PS-O, LL, BM, and SM contributed to the writing and editing of this manuscript.

## Conflict of Interest Statement

The authors declare that the research was conducted in the absence of any commercial or financial relationships that could be construed as a potential conflict of interest.

## Footnotes

1 https://theBioGrid.org/

2 http://www.hprd.org/

3 https://www.ebi.ac.uk/intact/

4 https://string-db.org/

5 http://dip.mbi.ucla.edu/dip/

6 http://mips.helmholtz-muenchen.de/corum/

7 https://geneindexer.com/

## Abbreviations

α_2A_-AR: α_2A_-adrenergic receptor
AD: Alzheimer’s disease
AT1aR: angiotensin II type 1A receptor
Ang II: angiotensin II
β2AR: β2-adrenergic receptor
cAMP: cyclic adenosine monophosphate
DDR: DNA damage response
GLP-1: glucagon-like peptide 1
GLP-1R: GLP1 receptor
GPCR: G protein coupled receptor
GRK: G protein coupled receptor kinase
GTP: guanosine triphosphate
IRS1: insulin receptor substrate-1
MAPK: mitogen activated protein kinase
NF-κB: nuclear factor-κB
PK: protein kinase
PTH: parathyroid hormone
PTH1R: parathyroid hormone 1 receptor
ROS: reactive oxygen species
SII: Sar1, Ile4, Ile8-AngII
T2DM: type II diabetes mellitus
