# Supplementary material for: β-Arrestin Based Receptor Signaling Paradigms: Potential Therapeutic Targets for Complex Age-Related Disorders
Source: Front Pharmacol. 2018 Nov 28;9:1369. doi: 10.3389/fphar.2018.01369 (PMC6280185; doi:10.3389/fphar.2018.01369)
Supplement: Supplementary file 4 [file Table_4.DOCX]

Supplementary Material

β-arrestin-based receptor signaling paradigms: potential therapeutic targets for complex age-related disorders First Author*, Co-Author, Co-Author

*** Correspondence:** Stuart Maudsley: [Stuart.Maudsley@uantwerpen.vib.be](mailto:Stuart.Maudsley@uantwerpen.vib.be)

# Supplementary Figures and Tables

Contents:

Table S1: Interactome Metadata extracted for β-arrestin1

Table S2: Interactome Metadata extracted for β-arrestin2

Table S3: Interrogation of interactome metadata for β-arrestin1 and 2 using GeneIndexer

**Table S1: *Interactome Metadata extracted for β-arrestin1***

| Gene Name | Source 1 | Source 2 | Source 3 | Source 4 | Source 5 | Source 6 | Source 7 |
| --- | --- | --- | --- | --- | --- | --- | --- |
| 5HT4R |  |  | IntAct |  |  |  |  |
| ACACA | BioGrid |  | IntAct |  |  |  |  |
| ACIN1 | BioGrid |  |  |  |  |  |  |
| ACM1 |  |  | IntAct |  |  |  |  |
| ACTA1 | BioGrid |  |  |  |  |  |  |
| ACTB |  |  | IntAct |  |  |  |  |
| ACTBL |  |  | IntAct |  |  |  |  |
| ACTBM |  |  | IntAct |  |  |  |  |
| ACTS |  |  | IntAct |  |  |  |  |
| ADH6 | BioGrid |  | IntAct |  |  |  |  |
| ADRA1B |  |  |  |  | STRING |  |  |
| ADRB1 | BioGrid | HPRD | IntAct |  |  | DIP |  |
| ADRB2 |  | HPRD |  |  | STRING |  |  |
| ADRBK1 | BioGrid |  |  |  | STRING |  |  |
| ADRBK2 |  |  |  |  | STRING |  |  |
| AGTR1 | BioGrid | HPRD | IntAct | MINT | STRING |  |  |
| AGTR1A | BioGrid |  |  |  |  |  |  |
| AINX |  |  | IntAct |  |  |  |  |
| AKT1 | BioGrid |  | IntAct |  |  |  |  |
| ALDH4A1 | BioGrid |  |  |  |  |  |  |
| ALYREF | BioGrid |  |  |  |  |  |  |
| ANM5 |  |  | IntAct |  |  |  |  |
| ANXA2 | BioGrid |  | IntAct |  |  |  |  |
| AP1B1 |  |  |  |  | STRING |  |  |
| AP2A1 | BioGrid |  |  |  | STRING |  |  |
| AP2B1 |  | HPRD |  |  | STRING |  |  |
| AP3B1 | BioGrid |  | IntAct |  | STRING |  |  |
| AP3D1 | BioGrid |  | IntAct |  |  |  |  |
| AP4B1 |  |  |  |  | STRING |  |  |
| APLP1 | BioGrid |  | IntAct |  |  |  |  |
| ARF6 | BioGrid | HPRD |  |  | STRING |  |  |
| ARHGAP32 | BioGrid |  |  |  |  |  |  |
| ARHGEF18 |  |  |  |  |  |  | CORUM |
| ARPC5 | BioGrid |  | IntAct |  |  |  |  |
| ARR3 | BioGrid |  |  |  |  |  |  |
| ARRB1 |  |  | IntAct |  |  |  |  |
| ARRB2 | BioGrid |  | IntAct |  | STRING |  |  |
| ARRC |  |  | IntAct |  |  |  |  |
| ARRDC1 | BioGrid |  |  |  |  |  |  |
| ARRDC3 | BioGrid |  |  |  |  |  |  |
| ARRDC4 | BioGrid |  |  |  |  |  |  |
| ARRS |  |  | IntAct |  |  |  |  |
| ASK1 |  |  |  | MINT |  |  |  |
| ATP5A1 | BioGrid |  |  |  |  |  |  |
| ATP5B | BioGrid |  |  |  |  |  |  |
| ATPA |  |  | IntAct |  |  |  |  |
| ATPB |  |  | IntAct |  |  |  |  |
| ATR | BioGrid |  | IntAct |  |  |  |  |
| AVPR2 |  |  |  |  | STRING |  |  |
| AXIN1 | BioGrid |  | IntAct |  |  | DIP |  |
| BACH2 | BioGrid |  | IntAct |  |  |  |  |
| BAG1 | BioGrid |  | IntAct |  |  |  |  |
| BCLAF1 | BioGrid |  |  |  |  |  |  |
| BCLF1 |  |  | IntAct |  |  |  |  |
| BIP |  |  | IntAct |  |  |  |  |
| BOP1 | BioGrid |  | IntAct |  |  |  |  |
| BRPF1 | BioGrid |  |  |  |  |  |  |
| BTK | BioGrid |  | IntAct |  |  |  |  |
| C5AR1 | BioGrid |  |  |  |  |  |  |
| CALD1 | BioGrid |  | IntAct |  |  |  |  |
| CALM |  |  | IntAct |  |  |  |  |
| CALM3 | BioGrid |  |  |  |  |  |  |
| CASB |  |  | IntAct |  |  |  |  |
| CBPA1 |  |  | IntAct |  |  |  |  |
| CCL14 | BioGrid |  | IntAct |  |  |  |  |
| CCR2 |  | HPRD |  |  |  |  |  |
| CCR5 | BioGrid | HPRD |  |  | STRING |  |  |
| CD247 |  |  |  | MINT |  |  |  |
| CD3EAP | BioGrid |  |  |  |  |  |  |
| CD3Z |  |  | IntAct |  |  |  |  |
| CDC42 | BioGrid |  | IntAct |  |  |  |  |
| CFL1 | BioGrid |  |  |  |  |  |  |
| CHD1 | BioGrid |  |  |  |  |  |  |
| CHRM1 |  |  |  |  | STRING |  |  |
| CKAP4 | BioGrid |  | IntAct |  |  |  |  |
| CLCA |  |  | IntAct |  |  |  |  |
| CLH1 |  |  | IntAct |  |  |  |  |
| CLTA | BioGrid |  |  |  |  |  |  |
| CLTC | BioGrid |  |  | MINT | STRING |  |  |
| CLTCL1 |  | HPRD |  |  | STRING |  |  |
| CMBL | BioGrid |  | IntAct |  |  |  |  |
| CNGA3 | BioGrid |  | IntAct |  |  |  |  |
| COF1 |  |  | IntAct |  |  |  |  |
| CPA1 | BioGrid |  |  |  |  |  |  |
| CPNE8 | BioGrid |  | IntAct |  |  |  |  |
| CSK | BioGrid | HPRD |  |  | STRING |  |  |
| CSK21 |  |  | IntAct |  |  |  |  |
| CSN2 | BioGrid |  |  |  |  |  |  |
| CSNK2A1 | BioGrid |  |  |  |  |  |  |
| CTTN | BioGrid |  |  |  |  |  |  |
| CUL3 | BioGrid |  |  |  |  |  |  |
| CXCL12 |  |  |  |  | STRING |  |  |
| CXCR2 | BioGrid |  |  |  | STRING |  |  |
| CXCR4 |  |  |  |  | STRING |  |  |
| CYTH1 | BioGrid |  |  |  |  |  |  |
| CYTH2 | BioGrid | HPRD |  |  | STRING |  |  |
| DCAF5 | BioGrid |  |  |  |  |  |  |
| DDX27 | BioGrid |  | IntAct |  |  |  |  |
| DGKE | BioGrid |  | IntAct |  |  |  |  |
| DGKG | BioGrid |  | IntAct |  |  |  |  |
| DGKH | BioGrid |  | IntAct |  |  |  |  |
| DGKZ | BioGrid |  | IntAct |  |  |  |  |
| DHRS2 | BioGrid |  | IntAct |  |  |  |  |
| DKC1 | BioGrid |  | IntAct |  |  |  |  |
| DMD | BioGrid |  | IntAct |  |  |  |  |
| DNAH3 | BioGrid |  |  |  |  |  |  |
| DNM1 | BioGrid |  |  |  |  |  |  |
| DOCK4 | BioGrid |  |  |  |  |  |  |
| DPY30 | BioGrid |  | IntAct |  |  |  |  |
| DRD4 | BioGrid |  |  |  |  |  |  |
| DVL1 | BioGrid | HPRD |  |  |  |  |  |
| DVL2 | BioGrid | HPRD |  |  | STRING |  |  |
| DYH3 |  |  | IntAct |  |  |  |  |
| DYN1 |  |  | IntAct |  |  |  |  |
| EDNRA | BioGrid |  | IntAct |  |  | DIP |  |
| EEF1A2 | BioGrid |  |  |  |  |  |  |
| EF1A2 |  |  | IntAct |  |  |  |  |
| EGF |  |  |  |  | STRING |  |  |
| EGFR | BioGrid |  | IntAct |  | STRING |  |  |
| EIF3I | BioGrid |  | IntAct |  |  |  |  |
| EIF4B | BioGrid |  |  |  |  |  |  |
| ELMO2 | BioGrid |  |  |  |  |  |  |
| ENAH |  |  |  |  |  |  | CORUM |
| ENO1B |  |  | IntAct |  |  |  |  |
| EPS15 |  |  |  |  | STRING |  |  |
| ERK1 |  | HPRD |  |  |  |  |  |
| ERK2 |  | HPRD |  |  |  |  |  |
| FAS |  |  | IntAct |  |  |  |  |
| FASN | BioGrid |  |  |  |  |  |  |
| FGR |  | HPRD |  |  | STRING |  |  |
| FLNA | BioGrid | HPRD | IntAct |  |  |  |  |
| FPR1 |  |  |  |  | STRING |  |  |
| FZD4 |  |  |  |  | STRING |  |  |
| G3BP1 | BioGrid |  | IntAct |  |  |  |  |
| GELS |  |  | IntAct |  |  |  |  |
| GLP1R |  |  | IntAct |  |  | DIP |  |
| GNAQ |  |  | IntAct | MINT |  |  |  |
| GNAS |  |  |  | MINT | STRING |  |  |
| GNAS1 |  |  | IntAct |  |  |  |  |
| GNB1 | BioGrid |  |  |  | STRING |  |  |
| GNG2 | BioGrid |  |  |  |  |  |  |
| GNMT | BioGrid | HPRD | IntAct |  |  |  |  |
| GNMT3 |  |  | IntAct |  |  |  |  |
| GNMT4 |  |  | IntAct |  |  |  |  |
| GPR3 |  |  | IntAct |  |  |  |  |
| GPR50 | BioGrid |  |  |  |  |  |  |
| GRK2 |  | HPRD |  |  |  |  |  |
| GRK5 |  |  | IntAct | MINT | STRING |  |  |
| GRK6 |  |  |  |  | STRING |  |  |
| GRP75 |  |  | IntAct |  |  |  |  |
| GSK3B | BioGrid |  |  |  |  |  |  |
| GSN | BioGrid |  |  |  |  |  |  |
| H12 |  |  | IntAct |  |  |  |  |
| H1FX | BioGrid |  |  |  |  |  |  |
| H1X |  |  | IntAct |  |  |  |  |
| H2A1 |  |  | IntAct |  |  |  |  |
| H2A2B |  |  | IntAct |  |  |  |  |
| H2AFX | BioGrid |  |  |  |  |  |  |
| H2AX |  |  | IntAct |  |  |  |  |
| H2B1O |  |  | IntAct |  |  |  |  |
| H33 |  |  | IntAct |  |  |  |  |
| H3F3B | BioGrid |  |  |  |  |  |  |
| H4 |  |  | IntAct |  |  |  |  |
| HCK |  | HPRD |  |  | STRING |  |  |
| HCRTR1 | BioGrid |  |  |  |  |  |  |
| HDGFRP2 | BioGrid |  |  |  |  |  |  |
| HDGR2 |  |  | IntAct |  |  |  |  |
| HDLBP | BioGrid |  |  |  |  |  |  |
| HGS | BioGrid |  | IntAct | MINT |  |  |  |
| HIBCH | BioGrid |  |  |  |  |  |  |
| HIF1A |  |  |  |  |  |  |  |
| HIST1H1C | BioGrid |  | IntAct |  |  |  |  |
| HIST1H2AG | BioGrid |  |  |  |  |  |  |
| HIST1H2BO | BioGrid |  |  |  |  |  |  |
| HIST2H2AB | BioGrid |  |  |  |  |  |  |
| HIST2H4A | BioGrid |  |  |  |  |  |  |
| HNRH1 |  |  | IntAct |  |  |  |  |
| HNRNPA1 | BioGrid |  |  |  |  |  |  |
| HNRNPA3 | BioGrid |  |  |  |  |  |  |
| HNRNPH1 | BioGrid |  |  |  |  |  |  |
| HNRNPK | BioGrid |  |  |  |  |  |  |
| HNRNPL | BioGrid |  |  |  |  |  |  |
| HNRNPM | BioGrid |  |  |  |  |  |  |
| HNRNPU | BioGrid |  |  |  |  |  |  |
| HNRPK |  |  | IntAct |  |  |  |  |
| HNRPM |  |  | IntAct |  |  |  |  |
| HNRPU |  |  | IntAct |  |  |  |  |
| HS90A |  |  | IntAct |  |  |  |  |
| HSP71 |  |  | IntAct |  |  |  |  |
| HSP77 |  |  | IntAct |  |  |  |  |
| HSP7C |  |  | IntAct |  |  |  |  |
| HSP90AA1 | BioGrid |  |  |  |  |  |  |
| HSPA1B | BioGrid |  |  |  |  |  |  |
| HSPA5 | BioGrid |  |  |  |  |  |  |
| HSPA7 | BioGrid |  |  |  |  |  |  |
| HSPA8 | BioGrid |  |  |  |  |  |  |
| HSPA9 | BioGrid |  |  |  |  |  |  |
| HTR2C |  |  |  |  | STRING |  |  |
| IF2B2 |  |  | IntAct |  |  |  |  |
| IF4B |  |  | IntAct |  |  |  |  |
| IGF1R | BioGrid |  | IntAct |  |  |  |  |
| IGF2BP2 | BioGrid |  |  |  |  |  |  |
| IGHMBP2 | BioGrid |  |  |  |  |  |  |
| IKBA |  |  | IntAct |  |  |  |  |
| IKKA |  | HPRD |  |  |  |  |  |
| IKKB |  | HPRD |  |  |  |  |  |
| IL8ST |  | HPRD |  |  |  |  |  |
| ILK |  |  | IntAct | MINT |  |  |  |
| IMA4 |  |  | IntAct |  |  |  |  |
| INA | BioGrid |  |  |  |  |  |  |
| ITCH | BioGrid |  |  |  |  |  |  |
| JAK1 | BioGrid |  |  |  |  |  |  |
| JUN | BioGrid |  | IntAct |  |  |  |  |
| KBTBD8 | BioGrid |  |  |  |  |  |  |
| KCTD3 | BioGrid |  | IntAct |  |  |  |  |
| KHK | BioGrid |  | IntAct |  |  |  |  |
| KI26A |  |  | IntAct |  |  |  |  |
| KIF26A | BioGrid |  |  |  |  |  |  |
| KIF2C | BioGrid |  | IntAct |  |  |  |  |
| KIF3A | BioGrid |  | IntAct |  | STRING |  |  |
| KLHL12 | BioGrid |  |  |  |  |  |  |
| KPNA3 | BioGrid |  |  |  |  |  |  |
| KPYM |  |  | IntAct |  |  |  |  |
| KTN1 | BioGrid |  | IntAct |  |  |  |  |
| LAP2A |  |  | IntAct |  |  |  |  |
| LIMA1 | BioGrid |  | IntAct |  |  |  |  |
| LIMK1 | BioGrid |  |  |  |  |  |  |
| LRP1B | BioGrid |  | IntAct |  |  |  |  |
| LRP4 | BioGrid |  | IntAct |  |  |  |  |
| LYN |  |  |  |  | STRING |  |  |
| LYRM7 | BioGrid |  |  |  |  |  |  |
| M3K1 |  |  | IntAct |  |  |  |  |
| M3K5 |  |  | IntAct |  |  |  |  |
| MAP1B | BioGrid |  | IntAct |  |  |  |  |
| MAP2K1 |  |  |  |  | STRING |  |  |
| MAP2K3 | BioGrid |  |  |  |  |  |  |
| MAP2K4 | BioGrid |  |  |  |  |  |  |
| MAP3K1 | BioGrid |  |  |  |  |  |  |
| MAP3K5 | BioGrid |  |  |  |  |  |  |
| MAPK1 | BioGrid |  |  |  | STRING |  |  |
| MAPK10 | BioGrid |  |  | MINT |  |  |  |
| MAPK14 | BioGrid |  |  |  |  |  |  |
| MAPK3 | BioGrid |  |  |  | STRING |  |  |
| MAPK9 | BioGrid |  |  |  |  |  |  |
| MDM2 | BioGrid |  | IntAct |  |  | DIP |  |
| MEP50 |  |  | IntAct |  |  |  |  |
| MK09 |  |  | IntAct |  |  |  |  |
| MK10 |  |  | IntAct |  |  |  |  |
| MKK4 |  |  |  | MINT |  |  |  |
| ML12B |  |  | IntAct |  |  |  |  |
| MLNR | BioGrid |  |  |  |  |  |  |
| MP2K4 |  |  | IntAct |  |  |  |  |
| MPRIP | BioGrid |  | IntAct |  |  |  |  |
| MT1A |  |  | IntAct | MINT |  |  |  |
| MYH1 | BioGrid |  | IntAct |  |  |  |  |
| MYH9 | BioGrid |  | IntAct |  |  |  |  |
| MYL12B | BioGrid |  |  |  |  |  |  |
| MYL6 | BioGrid |  | IntAct |  |  |  |  |
| MYO1C | BioGrid |  | IntAct |  |  |  |  |
| NAA10 | BioGrid |  | IntAct |  |  |  |  |
| NAA15 | BioGrid |  |  |  |  |  |  |
| NCL | BioGrid |  |  |  |  |  |  |
| NDUS1 |  |  | IntAct |  |  |  |  |
| NEDD4 | BioGrid | HPRD |  |  |  |  |  |
| NEK6 | BioGrid |  | IntAct |  |  |  |  |
| NFKBIA | BioGrid |  |  |  |  |  |  |
| NIK |  | HPRD |  |  |  |  |  |
| NOLC1 | BioGrid |  | IntAct |  |  |  |  |
| NOP10 | BioGrid |  | IntAct |  |  |  |  |
| NOTCH1 | BioGrid |  |  |  | STRING |  |  |
| NPM |  |  | IntAct |  |  |  |  |
| NPM1 | BioGrid |  |  |  |  |  |  |
| NSF | BioGrid | HPRD |  |  |  |  |  |
| NUCL |  |  | IntAct |  |  |  |  |
| OPRD1 | BioGrid |  |  |  |  |  |  |
| OPRM1 |  | HPRD |  |  |  |  |  |
| P3C2A |  |  | IntAct |  |  |  |  |
| P4HB | BioGrid |  |  |  |  |  |  |
| P53 |  |  | IntAct |  |  |  |  |
| P85B |  |  | IntAct |  |  |  |  |
| PARK2 | BioGrid |  |  |  |  |  |  |
| PDE4D | BioGrid |  |  |  |  |  |  |
| PDIA1 |  |  | IntAct |  |  |  |  |
| PDXP | BioGrid |  |  |  |  |  |  |
| PE2R2 |  |  | IntAct |  |  |  |  |
| PE2R4 |  |  | IntAct |  |  |  |  |
| PES1 | BioGrid |  |  |  |  |  |  |
| PESC |  |  | IntAct |  |  |  |  |
| PFKFB3 | BioGrid |  | IntAct | MINT | STRING | DIP |  |
| PIK3C2A | BioGrid |  |  |  |  |  |  |
| PIK3R2 | BioGrid |  |  |  |  |  |  |
| PKM | BioGrid |  |  |  |  |  |  |
| PLA1A |  |  | IntAct |  |  |  |  |
| POLR1A | BioGrid |  |  |  |  |  |  |
| POLR1C | BioGrid |  |  |  |  |  |  |
| POLR1E | BioGrid |  |  |  |  |  |  |
| POLR2E | BioGrid |  |  |  |  |  |  |
| POT1 | BioGrid |  |  |  |  |  |  |
| POTE1 |  |  | IntAct |  |  |  |  |
| PPIA |  |  | IntAct |  |  |  |  |
| PPM1A | BioGrid |  | IntAct |  |  |  |  |
| PPM1B | BioGrid |  | IntAct |  |  |  |  |
| PPP2CA |  |  |  |  | STRING |  |  |
| PRMT5 | BioGrid |  |  |  |  |  |  |
| PRP4B |  |  | IntAct |  |  |  |  |
| PRPF4 | BioGrid |  |  |  |  |  |  |
| PTAFR | BioGrid |  |  |  |  |  |  |
| PTC2 |  |  | IntAct |  |  |  |  |
| PTCH2 | BioGrid |  |  |  |  |  |  |
| PTGER4 | BioGrid |  |  |  |  | DIP |  |
| PTH1R |  | HPRD |  |  |  |  |  |
| PTHLH | BioGrid | HPRD |  |  |  |  |  |
| Q3MIH3 |  |  | IntAct |  |  |  |  |
| Q5RKT7 |  |  | IntAct |  |  |  |  |
| Q5U5U6 |  |  | IntAct |  |  |  |  |
| Q5UGI3 |  |  | IntAct |  |  |  |  |
| Q6ZSQ4 |  |  | IntAct |  |  |  |  |
| Q7Z3R3 |  |  | IntAct |  |  |  |  |
| Q8TBK5 |  |  | IntAct |  |  |  |  |
| Q96K27 |  |  | IntAct |  |  |  |  |
| RAB11FIP5 | BioGrid |  |  |  |  |  |  |
| RAB1A | BioGrid |  | IntAct |  |  |  |  |
| RAB5A |  |  |  |  | STRING |  |  |
| RAF1 |  |  |  |  | STRING |  |  |
| RALGDS | BioGrid | HPRD |  |  |  |  |  |
| RANB9 |  |  | IntAct |  |  |  |  |
| RANBP9 | BioGrid |  |  |  |  |  |  |
| RBM10 | BioGrid |  | IntAct |  |  |  |  |
| RFIP5 |  |  | IntAct |  |  |  |  |
| RGS3 | BioGrid |  | IntAct |  |  |  |  |
| RHG32 |  |  | IntAct |  |  |  |  |
| RHO |  |  |  |  | STRING |  |  |
| RL15 |  |  | IntAct |  |  |  |  |
| RL22 |  |  | IntAct |  |  |  |  |
| RL3 |  |  | IntAct |  |  |  |  |
| RL7A |  |  | IntAct |  |  |  |  |
| RL7L |  |  | IntAct |  |  |  |  |
| ROA1 |  |  | IntAct |  |  |  |  |
| ROA3 |  |  | IntAct |  |  |  |  |
| ROCK1 | BioGrid |  | IntAct |  |  |  |  |
| RPA1 |  |  | IntAct |  |  |  |  |
| RPA34 |  |  | IntAct |  |  |  |  |
| RPA49 |  |  | IntAct |  |  |  |  |
| RPAB1 |  |  | IntAct |  |  |  |  |
| RPAC1 |  |  | IntAct |  |  |  |  |
| RPL15 | BioGrid |  |  |  |  |  |  |
| RPL22 | BioGrid |  |  |  |  |  |  |
| RPL3 | BioGrid |  |  |  |  |  |  |
| RPL6 | BioGrid |  |  |  |  |  |  |
| RPL7A | BioGrid |  |  |  |  |  |  |
| RPL7L1 | BioGrid |  |  |  |  |  |  |
| RPS17 | BioGrid |  |  |  |  |  |  |
| RPS2 | BioGrid |  |  |  |  |  |  |
| RPS27A | BioGrid |  |  |  |  |  |  |
| RPS3A | BioGrid |  |  |  |  |  |  |
| RPS8 | BioGrid |  |  |  |  |  |  |
| RS17 |  |  | IntAct |  |  |  |  |
| RS2 |  |  | IntAct |  |  |  |  |
| RS3A |  |  | IntAct |  |  |  |  |
| RS8 |  |  | IntAct |  |  |  |  |
| RTF1 | BioGrid |  | IntAct |  |  |  |  |
| S100A9 | BioGrid |  |  |  |  |  |  |
| S10A9 |  |  | IntAct |  |  |  |  |
| SAG | BioGrid |  |  |  |  |  |  |
| SCYL2 | BioGrid |  | IntAct |  |  |  |  |
| SDC3 | BioGrid |  | IntAct |  |  |  |  |
| SF3B2 | BioGrid |  | IntAct |  |  |  |  |
| SLC2A14 | BioGrid |  |  |  |  |  |  |
| SLC9A1 | BioGrid |  |  |  |  |  |  |
| SLC9A5 | BioGrid | HPRD |  |  |  |  |  |
| SMD1 |  |  | IntAct |  |  |  |  |
| SMO |  |  | IntAct | MINT | STRING |  |  |
| SNCA | BioGrid |  |  |  |  |  |  |
| SNRPD1 | BioGrid |  |  |  |  |  |  |
| SPIN1 | BioGrid |  | IntAct |  |  |  |  |
| SPTAN1 | BioGrid |  |  |  |  |  |  |
| SPTN1 |  |  | IntAct |  |  |  |  |
| SRBP2 |  |  | IntAct |  |  |  |  |
| SRC | BioGrid |  | IntAct |  | STRING | DIP |  |
| SRC8 |  |  | IntAct |  |  |  |  |
| SREBF2 | BioGrid |  |  |  |  |  |  |
| SRRM2 | BioGrid |  | IntAct |  |  |  |  |
| ST38L |  |  | IntAct |  |  |  |  |
| STAM | BioGrid |  |  |  |  |  |  |
| STK38 | BioGrid |  | IntAct |  |  |  |  |
| STK38L | BioGrid |  |  |  |  |  |  |
| STUB1 | BioGrid |  |  |  |  |  |  |
| STXB5 |  |  | IntAct |  |  |  |  |
| STXBP5 | BioGrid |  |  |  |  |  |  |
| TACR1 |  |  |  |  | STRING |  |  |
| TBA1C |  |  | IntAct |  |  |  |  |
| TBA3C |  |  | IntAct |  |  |  |  |
| TBA4A |  |  | IntAct |  |  |  |  |
| TBB2A |  |  | IntAct |  |  |  |  |
| TBB5 |  |  | IntAct |  |  |  |  |
| TCOF |  |  | IntAct |  |  |  |  |
| TCOF1 | BioGrid |  |  |  |  |  |  |
| TF |  |  |  |  | STRING |  |  |
| THOC4 |  |  | IntAct |  |  |  |  |
| THRAP3 | BioGrid |  |  |  |  |  |  |
| TITIN |  |  | IntAct |  |  |  |  |
| TMOD3 | BioGrid |  | IntAct |  |  |  |  |
| TMPO | BioGrid |  |  |  |  |  |  |
| TP53 |  |  |  |  |  | DIP |  |
| TPM4 | BioGrid |  | IntAct |  |  |  |  |
| TR150 |  |  | IntAct |  |  |  |  |
| TRAF6 | BioGrid |  | IntAct |  |  |  |  |
| TRBV7-9 |  |  |  | MINT |  |  |  |
| TRHR | BioGrid |  |  |  |  |  |  |
| TSHR |  | HPRD |  |  |  |  |  |
| TTN | BioGrid |  |  |  |  |  |  |
| TUBA1C | BioGrid |  |  |  |  |  |  |
| TUBA3C | BioGrid |  |  |  |  |  |  |
| TUBA4A | BioGrid |  |  |  |  |  |  |
| TUBB | BioGrid |  |  |  |  |  |  |
| TUBB2A | BioGrid |  |  |  |  |  |  |
| TVB79 |  |  | IntAct |  |  |  |  |
| TYY1 |  |  | IntAct |  |  |  |  |
| UBA52 | BioGrid |  |  |  |  |  |  |
| UBB | BioGrid |  |  |  |  |  |  |
| UBC | BioGrid |  |  |  |  |  |  |
| UBP24 |  |  | IntAct |  |  |  |  |
| USP24 | BioGrid |  |  |  |  |  |  |
| USP33 | BioGrid |  |  |  | STRING |  |  |
| VIGLN |  |  | IntAct |  |  |  |  |
| VIM | BioGrid |  |  |  |  |  |  |
| VIME |  |  | IntAct |  |  |  |  |
| WDR77 | BioGrid |  |  |  |  |  |  |
| XRCC5 | BioGrid |  | IntAct |  |  |  |  |
| YBOX1 |  |  | IntAct |  |  |  |  |
| YBX1 | BioGrid |  |  |  |  |  |  |
| YES |  |  | IntAct |  |  |  |  |
| YES1 | BioGrid |  |  |  |  |  |  |
| YWHAB | BioGrid |  | IntAct |  |  |  |  |
| YWHAE | BioGrid |  | IntAct |  |  |  |  |
| YWHAG | BioGrid |  | IntAct |  |  |  |  |
| YWHAH | BioGrid |  | IntAct |  |  |  |  |
| YWHAQ | BioGrid |  | IntAct |  |  |  |  |
| YWHAZ | BioGrid |  | IntAct |  |  |  |  |
| YY1 | BioGrid |  |  |  |  |  |  |
| ZBT43 |  |  | IntAct |  |  |  |  |
| ZBTB43 | BioGrid |  |  |  |  |  |  |
| ZRAB2 |  |  | IntAct |  |  |  |  |
| ZRANB | BioGrid |  |  |  |  |  |  |
| ZYX | BioGrid |  | IntAct |  |  |  |  |

**Table S2: *Interactome Metadata extracted for β-arrestin2***

| Gene Name | Source 1 | Source 2 | Source 3 | Source 4 | Source 5 | Source 6 | Source 7 |
| --- | --- | --- | --- | --- | --- | --- | --- |
| AARSD1 | BioGrid |  |  |  |  |  |  |
| ACM1 |  |  | IntAct |  |  |  |  |
| ACTB | BioGrid |  | IntAct |  |  |  |  |
| ACTBL |  |  | IntAct |  |  |  |  |
| ACTBM |  |  | IntAct |  |  |  |  |
| ACTC |  |  | IntAct |  |  |  |  |
| ACTC1 | BioGrid |  |  |  |  |  |  |
| ACTG2 | BioGrid |  |  |  |  |  |  |
| ACTH |  |  | IntAct |  |  |  |  |
| ADCY2 |  |  |  |  |  |  | CORUM |
| ADRB1 | BioGrid |  | IntAct |  |  | DIP |  |
| ADRB2 | BioGrid | HPRD | IntAct | MINT | STRING | DIP |  |
| ADRBK1 |  |  |  |  | STRING |  |  |
| ADRBK2 |  |  |  |  | STRING |  |  |
| AFAD |  |  | IntAct |  |  |  |  |
| AFF4 | BioGrid |  |  |  |  |  |  |
| AGTR1 | BioGrid | HPRD |  |  | STRING |  |  |
| AHCYL1 | BioGrid |  |  |  |  |  |  |
| AHCYL2 | BioGrid |  |  |  |  |  |  |
| AKAP12 |  | HPRD |  |  |  |  |  |
| AKAP5 |  |  |  |  |  |  | CORUM |
| AKT1 | BioGrid |  | IntAct |  |  | DIP |  |
| AKT2 |  |  | IntAct |  |  | DIP |  |
| ALYREF | BioGrid |  |  |  |  |  |  |
| ANKRD11 | BioGrid |  |  |  |  |  |  |
| ANM1 |  |  | IntAct |  |  |  |  |
| ANM5 |  |  | IntAct |  |  |  |  |
| ANR11 |  |  | IntAct |  |  |  |  |
| ANXA2 | BioGrid |  | IntAct |  |  |  |  |
| AP1B1 | BioGrid | HPRD |  |  |  |  |  |
| AP2A1 | BioGrid |  | IntAct |  |  |  |  |
| AP2A2 | BioGrid |  | IntAct |  |  |  |  |
| AP2B1 | BioGrid |  | IntAct |  | STRING |  |  |
| AP2M1 | BioGrid | HPRD |  |  | STRING |  |  |
| AP3B1 | BioGrid |  | IntAct |  |  |  |  |
| AP3B2 | BioGrid |  | IntAct |  |  |  |  |
| AP3D1 | BioGrid |  | IntAct |  |  |  |  |
| ARF6 | BioGrid | HPRD |  |  | STRING |  |  |
| ARHG6 |  |  | IntAct |  |  |  |  |
| ARHGAP17 | BioGrid |  |  |  |  |  |  |
| ARHGAP21 | BioGrid |  |  |  |  |  |  |
| ARHGAP22 | BioGrid |  |  |  |  |  |  |
| ARHGB |  |  | IntAct |  |  |  |  |
| ARHGC |  |  | IntAct |  |  |  |  |
| ARHGEF11 | BioGrid |  |  |  |  |  |  |
| ARHGEF12 | BioGrid |  |  |  |  |  |  |
| ARHGEF6 | BioGrid |  |  |  |  |  |  |
| ARP5L |  |  | IntAct |  |  |  |  |
| ARPC5L | BioGrid |  |  |  |  |  |  |
| ARR3 | BioGrid |  |  |  |  |  |  |
| ARRB1 | BioGrid |  | IntAct |  | STRING |  |  |
| ARRB2 |  |  | IntAct |  |  |  |  |
| ARRC |  |  | IntAct |  |  |  |  |
| ARRDC3 | BioGrid |  |  |  |  |  |  |
| ARRDC4 | BioGrid |  |  |  |  |  |  |
| ARRS |  |  | IntAct |  |  |  |  |
| ASK1 |  | HPRD |  | MINT |  |  | CORUM |
| AVPR2 | BioGrid | HPRD |  |  | STRING |  |  |
| BAI1 | BioGrid |  |  |  |  |  |  |
| BCLAF1 | BioGrid |  |  |  |  |  |  |
| BCLF1 |  |  | IntAct |  |  |  |  |
| BIP |  |  | IntAct |  |  |  |  |
| BOLA2 | BioGrid |  | IntAct |  |  |  |  |
| BOLA2B | BioGrid |  |  |  |  |  |  |
| BOP1 | BioGrid |  | IntAct |  |  |  |  |
| BRPF1 | BioGrid |  |  |  |  |  |  |
| BSN | BioGrid |  | IntAct |  |  |  |  |
| C16ORF71 | BioGrid |  |  |  |  |  |  |
| C18ORF25 | BioGrid |  |  |  |  |  |  |
| C1QBP | BioGrid |  | IntAct |  |  |  |  |
| C5AR1 | BioGrid |  |  |  |  |  |  |
| CALCRL |  | HPRD |  |  |  |  |  |
| CALM |  |  | IntAct |  |  |  |  |
| CALM3 | BioGrid |  |  |  |  |  |  |
| CAMK2D | BioGrid |  |  |  |  |  |  |
| CAN1 |  |  | IntAct |  |  |  |  |
| CAPN1 | BioGrid |  |  |  |  |  |  |
| CAPZA1 | BioGrid |  |  |  |  |  |  |
| CAPZA2 | BioGrid |  |  |  |  |  |  |
| CASB |  |  | IntAct |  |  |  |  |
| CAZA1 |  |  | IntAct |  |  |  |  |
| CAZA2 |  |  | IntAct |  |  |  |  |
| CCDC102B | BioGrid |  |  |  |  |  |  |
| CCT6A | BioGrid |  |  |  |  |  |  |
| CDC42 | BioGrid |  | IntAct |  |  |  |  |
| CDK13 | BioGrid |  | IntAct |  |  |  |  |
| CDK3 | BioGrid |  | IntAct |  |  |  |  |
| CDK4 | BioGrid |  | IntAct |  |  |  |  |
| CDK7 | BioGrid |  | IntAct |  |  |  |  |
| CE170 |  |  | IntAct |  |  |  |  |
| CENPB | BioGrid |  |  |  |  |  |  |
| CENPF | BioGrid |  | IntAct |  |  |  |  |
| CENPM | BioGrid |  |  |  |  |  |  |
| CEP170 | BioGrid |  |  |  |  |  |  |
| CFL1 | BioGrid |  |  |  |  |  |  |
| CHUK | BioGrid |  |  |  |  |  |  |
| CKA1 |  | HPRD |  |  |  |  |  |
| CKA2 |  | HPRD |  |  |  |  |  |
| CLCA |  |  | IntAct |  |  |  |  |
| CLCB |  |  | IntAct |  |  |  |  |
| CLIC1 | BioGrid |  | IntAct |  |  |  |  |
| CLNS1A | BioGrid |  |  |  |  |  |  |
| CLTA | BioGrid |  |  |  |  |  |  |
| CLTB | BioGrid |  |  |  |  |  |  |
| CLTC | BioGrid | HPRD |  |  | STRING |  |  |
| CLTCL1 |  | HPRD |  |  | STRING |  |  |
| COF1 |  |  | IntAct |  |  |  |  |
| COPRS | BioGrid |  | IntAct |  |  |  |  |
| CPNE1 | BioGrid |  | IntAct |  |  |  |  |
| CSK | BioGrid |  |  |  | STRING |  |  |
| CSK21 |  |  | IntAct |  |  |  |  |
| CSN2 | BioGrid |  |  |  |  |  |  |
| CSNK1A1L | BioGrid |  |  |  |  |  |  |
| CSNK2A1 | BioGrid |  |  |  |  |  |  |
| CSNK2A2 | BioGrid |  |  |  |  |  |  |
| CTNA1 |  |  | IntAct |  |  |  |  |
| CTND1 |  |  | IntAct |  |  |  |  |
| CTNNA1 | BioGrid |  |  |  |  |  |  |
| CTNND1 | BioGrid |  |  |  |  |  |  |
| CTPS1 | BioGrid |  |  |  |  |  |  |
| CTTN | BioGrid |  |  |  | STRING |  |  |
| CUL3 | BioGrid |  |  |  |  |  |  |
| CUL5 | BioGrid |  | IntAct |  |  |  |  |
| CXCR2 |  |  |  |  | STRING |  |  |
| CXCR4 |  | HPRD |  |  | STRING |  |  |
| CYTH1 | BioGrid |  |  |  |  |  |  |
| CYTH2 | BioGrid |  |  |  | STRING |  |  |
| CYTIP |  | HPRD |  |  |  |  |  |
| DBN1 | BioGrid |  |  |  |  |  |  |
| DCD | BioGrid |  | IntAct |  |  |  |  |
| DDX1 | BioGrid |  | IntAct |  |  |  |  |
| DDX27 | BioGrid |  | IntAct |  |  |  |  |
| DDX3X | BioGrid |  | IntAct |  |  |  |  |
| DDX5 | BioGrid |  | IntAct |  |  |  |  |
| DGKE | BioGrid |  | IntAct |  |  |  |  |
| DGKZ | BioGrid |  | IntAct |  |  |  |  |
| DHX15 | BioGrid |  | IntAct |  |  |  |  |
| DIEXF | BioGrid |  |  |  |  |  |  |
| DKC1 | BioGrid |  | IntAct |  |  |  |  |
| DRD2 | BioGrid |  |  |  | STRING |  |  |
| DRD4 | BioGrid |  |  |  |  |  |  |
| DREB |  |  | IntAct |  |  |  |  |
| DVL2 |  | HPRD |  |  | STRING |  |  |
| DYHC1 |  |  | IntAct |  |  |  |  |
| DYNC1H1 | BioGrid |  |  |  |  |  |  |
| E2AK4 |  |  | IntAct |  |  |  |  |
| E41L3 |  |  | IntAct |  |  |  |  |
| EDNRA | BioGrid |  | IntAct |  |  | DIP |  |
| EEF1A1 | BioGrid |  |  |  |  |  |  |
| EEF1A2 | BioGrid |  |  |  |  |  |  |
| EEF2 | BioGrid |  |  |  |  |  |  |
| EF1A1 |  |  | IntAct |  |  |  |  |
| EF1A2 |  |  | IntAct |  |  |  |  |
| EF2 |  |  | IntAct |  |  |  |  |
| EFTUD2 | BioGrid |  |  |  |  |  |  |
| EGF |  |  |  |  | STRING |  |  |
| EGFR | BioGrid |  | IntAct |  | STRING |  |  |
| EGLN |  |  | IntAct |  |  |  |  |
| EIF1AD | BioGrid |  |  |  |  |  |  |
| EIF2AK4 | BioGrid |  |  |  |  |  |  |
| EIF4B | BioGrid |  |  |  |  |  |  |
| EMILIN1 | BioGrid |  |  |  |  |  |  |
| ENG |  |  |  | MINT |  |  |  |
| EPB41L3 | BioGrid |  |  |  |  |  |  |
| EPB41L5 | BioGrid |  |  |  |  |  |  |
| ERH | BioGrid |  | IntAct |  |  |  |  |
| ERK1 |  | HPRD |  |  |  |  |  |
| ERK2 |  | HPRD |  |  |  |  |  |
| EVI2A | BioGrid |  |  |  |  |  |  |
| FAS |  |  | IntAct |  |  |  |  |
| FASN | BioGrid |  |  |  |  |  |  |
| FBL | BioGrid |  |  |  |  |  |  |
| FBRL |  |  | IntAct |  |  |  |  |
| FLNA | BioGrid | HPRD | IntAct |  |  |  |  |
| FLNB | BioGrid |  |  |  |  |  |  |
| FZD2 |  |  |  |  | STRING |  |  |
| FZD4 |  |  |  |  | STRING |  |  |
| G3BP2 | BioGrid |  | IntAct |  |  |  |  |
| G3P |  |  | IntAct |  |  |  |  |
| GAG-POL | BioGrid |  |  |  |  |  |  |
| GAPDH | BioGrid |  |  |  |  |  |  |
| GCG | BioGrid |  |  |  |  |  |  |
| GELS |  |  | IntAct |  |  |  |  |
| GLI1 |  |  |  |  | STRING |  |  |
| GLI2 |  |  |  |  | STRING |  |  |
| GLI3 |  |  |  |  | STRING |  |  |
| GNA11 |  |  |  |  | STRING |  |  |
| GNA15 |  |  |  |  | STRING |  |  |
| GNAQ |  |  |  |  | STRING |  |  |
| GPBP1L1 | BioGrid |  |  |  |  |  |  |
| GPR156 | BioGrid |  |  |  |  |  |  |
| GPR3 |  |  | IntAct |  |  |  |  |
| GPR37 | BioGrid |  |  |  |  |  |  |
| GPR55 | BioGrid |  |  |  |  |  |  |
| GPR56 | BioGrid |  |  |  |  |  |  |
| GRK2 |  | HPRD |  |  |  |  |  |
| GRK6 |  |  |  |  | STRING |  |  |
| GSN | BioGrid |  |  |  |  |  |  |
| H11 |  |  | IntAct |  |  |  |  |
| H12 |  |  | IntAct |  |  |  |  |
| H1FX | BioGrid |  |  |  |  |  |  |
| H1X |  |  | IntAct |  |  |  |  |
| H2A2B |  |  | IntAct |  |  |  |  |
| H2AFX | BioGrid |  |  |  |  |  |  |
| H2AX |  |  | IntAct |  |  |  |  |
| H2B1O |  |  | IntAct |  |  |  |  |
| HCRTR1 | BioGrid |  |  |  |  |  |  |
| HDAC2 | BioGrid |  | IntAct |  |  |  |  |
| HDGFRP2 | BioGrid |  |  |  |  |  |  |
| HDGR2 |  |  | IntAct |  |  |  |  |
| HGS | BioGrid |  | IntAct | MINT |  |  |  |
| HIPK3 | BioGrid | HPRD | IntAct | MINT |  |  |  |
| HIST1H1A | BioGrid |  |  |  |  |  |  |
| HIST1H1C | BioGrid |  |  |  |  |  |  |
| HIST1H2BO | BioGrid |  |  |  |  |  |  |
| HIST2H2AB | BioGrid |  |  |  |  |  |  |
| HNRH1 |  |  | IntAct |  |  |  |  |
| HNRH2 |  |  | IntAct |  |  |  |  |
| HNRNPA0 | BioGrid |  |  |  |  |  |  |
| HNRNPA1 | BioGrid |  |  |  |  |  |  |
| HNRNPA2B1 | BioGrid |  |  |  |  |  |  |
| HNRNPC | BioGrid |  |  |  |  |  |  |
| HNRNPD | BioGrid |  |  |  |  |  |  |
| HNRNPF | BioGrid |  |  |  |  |  |  |
| HNRNPH1 | BioGrid |  |  |  |  |  |  |
| HNRNPH2 | BioGrid |  |  |  |  |  |  |
| HNRNPK | BioGrid |  |  |  |  |  |  |
| HNRNPL | BioGrid |  |  |  |  |  |  |
| HNRNPM | BioGrid |  |  |  |  |  |  |
| HNRNPR | BioGrid |  |  |  |  |  |  |
| HNRNPU | BioGrid |  |  |  |  |  |  |
| HNRPC |  |  | IntAct |  |  |  |  |
| HNRPD |  |  | IntAct |  |  |  |  |
| HNRPF |  |  | IntAct |  |  |  |  |
| HNRPK |  |  | IntAct |  |  |  |  |
| HNRPM |  |  | IntAct |  |  |  |  |
| HNRPR |  |  | IntAct |  |  |  |  |
| HNRPU |  |  | IntAct |  |  |  |  |
| HS71L |  |  | IntAct |  |  |  |  |
| HS90B |  |  | IntAct |  |  |  |  |
| HSP71 |  |  | IntAct |  |  |  |  |
| HSP76 |  |  | IntAct |  |  |  |  |
| HSP7C |  |  | IntAct |  |  |  |  |
| HSP90AB1 | BioGrid |  |  |  |  |  |  |
| HSPA1B | BioGrid |  |  |  |  |  |  |
| HSPA1L | BioGrid |  |  |  |  |  |  |
| HSPA5 | BioGrid |  |  |  |  |  |  |
| HSPA6 | BioGrid |  |  |  |  |  |  |
| HSPA8 | BioGrid |  |  |  |  |  |  |
| HTATSF1 | BioGrid |  |  |  |  |  |  |
| HTR2A |  |  |  |  |  | DIP |  |
| HTR2C |  | HPRD |  |  |  |  |  |
| HTSF1 |  |  | IntAct |  |  |  |  |
| I6L957 |  |  | IntAct |  |  |  |  |
| ICLN |  |  | IntAct |  |  |  |  |
| IF2B1 |  |  | IntAct |  |  |  |  |
| IF4B |  |  | IntAct |  |  |  |  |
| IGF1R | BioGrid |  |  |  |  |  |  |
| IGF2BP1 | BioGrid |  |  |  |  |  |  |
| IGFALS | BioGrid |  |  |  |  |  |  |
| IGKV1-5 | BioGrid |  |  |  |  |  |  |
| IKKA |  | HPRD | IntAct |  |  |  |  |
| IKKB |  | HPRD |  |  |  |  |  |
| ILF3 | BioGrid |  | IntAct |  |  |  |  |
| ILK |  |  | IntAct | MINT |  |  |  |
| IMA3 |  |  | IntAct |  |  |  |  |
| IMA4 |  |  | IntAct |  |  |  |  |
| IMB1 |  |  | IntAct |  |  |  |  |
| INSR | BioGrid |  |  |  |  | DIP |  |
| ITCH | BioGrid | HPRD |  |  |  |  |  |
| JNK3 |  | HPRD |  |  |  |  | CORUM |
| JPH1 | BioGrid |  |  |  |  |  |  |
| KANK1 | BioGrid |  |  |  |  |  |  |
| KBTBD8 | BioGrid |  |  |  |  |  |  |
| KC1AL |  |  | IntAct |  |  |  |  |
| KCAB1 |  |  | IntAct |  |  |  |  |
| KCC2D |  |  | IntAct |  |  |  |  |
| KCNAB1 | BioGrid |  |  |  |  |  |  |
| KIF3A |  |  | IntAct | MINT | STRING |  |  |
| KLHL12 | BioGrid |  |  |  |  |  |  |
| KPB2 |  |  | IntAct |  |  |  |  |
| KPNA3 | BioGrid |  |  |  |  |  |  |
| KPNA4 | BioGrid |  |  |  |  |  |  |
| KPNB1 | BioGrid |  |  |  |  |  |  |
| KPYM |  |  | IntAct |  |  |  |  |
| LAP2A |  |  | IntAct |  |  |  |  |
| LBR | BioGrid |  | IntAct |  |  |  |  |
| LEO1 | BioGrid |  | IntAct |  |  |  |  |
| LHCGR | BioGrid |  |  |  | STRING |  |  |
| LIMA1 | BioGrid |  | IntAct |  |  |  |  |
| LIMK1 | BioGrid |  |  |  |  |  |  |
| LRP11 | BioGrid |  | IntAct |  |  |  |  |
| M3K1 |  |  | IntAct |  |  |  |  |
| M3K14 |  |  | IntAct |  |  |  |  |
| M3K5 |  |  | IntAct |  |  |  |  |
| M3K7 |  |  | IntAct |  |  |  |  |
| MANSC1 | BioGrid |  |  |  |  |  |  |
| MAP1B | BioGrid |  | IntAct |  |  |  |  |
| MAP2K1 | BioGrid |  |  |  | STRING |  |  |
| MAP2K2 |  |  |  |  | STRING |  |  |
| MAP2K4 | BioGrid | HPRD |  |  | STRING |  |  |
| MAP3K1 | BioGrid |  |  |  |  |  |  |
| MAP3K14 | BioGrid |  |  |  |  |  |  |
| MAP3K5 | BioGrid |  |  |  |  |  |  |
| MAP3K7 | BioGrid |  |  |  |  |  |  |
| MAPK1 | BioGrid |  |  |  | STRING |  |  |
| MAPK10 | BioGrid |  |  | MINT |  |  |  |
| MAPK14 |  |  |  |  | STRING |  |  |
| MAPK3 | BioGrid |  |  |  | STRING |  |  |
| MAPK9 | BioGrid |  |  |  |  |  |  |
| MAS1 | BioGrid |  |  |  |  |  |  |
| MCM3 | BioGrid |  | IntAct |  |  |  |  |
| MDM2 | BioGrid | HPRD | IntAct | MINT |  |  |  |
| MED27 |  | HPRD |  |  |  |  |  |
| MED4 | BioGrid |  |  |  |  |  |  |
| MED8 | BioGrid |  | IntAct |  |  |  |  |
| MEK1 |  | HPRD |  |  |  |  |  |
| MEP50 |  |  | IntAct |  |  |  |  |
| MK01 |  |  | IntAct |  |  |  |  |
| MK03 |  |  | IntAct |  |  |  |  |
| MK09 |  |  | IntAct |  |  |  |  |
| MK10 |  |  | IntAct |  |  |  |  |
| MKK4 |  |  |  | MINT |  |  | CORUM |
| MLLT1 | BioGrid |  |  |  |  |  |  |
| MLLT3 | BioGrid |  |  |  |  |  |  |
| MLLT4 | BioGrid |  |  |  |  |  |  |
| MLNR | BioGrid |  |  |  |  |  |  |
| MOB1A | BioGrid |  | IntAct |  |  |  |  |
| MP2K4 |  |  | IntAct |  |  |  |  |
| MRPL43 | BioGrid |  |  |  |  |  |  |
| MRPL44 | BioGrid |  |  |  |  |  |  |
| MYCD |  |  | IntAct |  |  |  |  |
| MYH10 | BioGrid |  | IntAct |  |  |  |  |
| MYH9 | BioGrid |  | IntAct |  |  |  |  |
| MYL6 | BioGrid |  | IntAct |  |  |  |  |
| MYO1C | BioGrid |  | IntAct |  |  |  |  |
| MYOCD | BioGrid |  |  |  |  |  |  |
| MYPT1 |  |  | IntAct |  |  |  |  |
| NAA10 | BioGrid |  | IntAct |  |  |  |  |
| NAA15 | BioGrid |  |  |  |  |  |  |
| NAP1L1 | BioGrid |  |  |  |  |  |  |
| NCL | BioGrid |  |  |  |  |  |  |
| NDUFS7 |  | HPRD |  |  |  |  |  |
| NDUS7 |  |  | IntAct |  |  |  |  |
| NEDD4 | BioGrid | HPRD | IntAct | MINT |  |  |  |
| NFKBIA | BioGrid |  |  |  | STRING |  |  |
| NFKBIL1 | BioGrid |  |  |  |  |  |  |
| NIK |  | HPRD |  |  |  |  |  |
| NINL | BioGrid |  | IntAct |  |  |  |  |
| NKTR | BioGrid |  | IntAct |  |  |  |  |
| NOLC1 | BioGrid |  | IntAct |  |  |  |  |
| NONO | BioGrid |  | IntAct |  |  |  |  |
| NOP10 | BioGrid |  | IntAct |  |  |  |  |
| NOP56 | BioGrid |  | IntAct |  |  |  |  |
| NP1L1 |  |  | IntAct |  |  |  |  |
| NPHS1 |  |  |  |  | STRING | DIP |  |
| NPM |  |  | IntAct |  |  |  |  |
| NPM1 | BioGrid |  |  |  |  |  |  |
| NSUN2 | BioGrid |  | IntAct |  |  |  |  |
| NTS |  | HPRD |  |  |  |  |  |
| NTSR1 |  | HPRD |  |  |  |  |  |
| NUCL |  |  | IntAct |  |  |  |  |
| OPRD1 | BioGrid |  |  |  |  |  |  |
| OPRM1 |  | HPRD |  |  |  |  |  |
| OXER1 | BioGrid |  |  |  |  |  |  |
| OXTR |  | HPRD |  |  |  |  |  |
| P3C2A |  |  | IntAct |  |  |  |  |
| P4HB | BioGrid |  |  |  |  |  |  |
| PABP1 |  |  | IntAct |  |  |  |  |
| PABP3 |  |  | IntAct |  |  |  |  |
| PABP4 |  |  | IntAct |  |  |  |  |
| PABPC1 | BioGrid |  |  |  |  |  |  |
| PABPC3 | BioGrid |  |  |  |  |  |  |
| PABPC4 | BioGrid |  |  |  |  |  |  |
| PAIRB |  |  | IntAct |  |  |  |  |
| PAK5 |  |  | IntAct |  |  |  |  |
| PAK7 | BioGrid |  |  |  |  |  |  |
| PARK2 | BioGrid |  |  |  |  |  |  |
| PDE4D | BioGrid |  | IntAct | MINT |  | DIP | CORUM |
| PDIA1 |  |  | IntAct |  |  |  |  |
| PE2R2 |  |  | IntAct |  |  |  |  |
| PEBP1 |  |  |  |  | STRING |  |  |
| PES1 | BioGrid |  |  |  |  |  |  |
| PESC |  |  | IntAct |  |  |  |  |
| PFKFB3 | BioGrid |  |  |  |  |  |  |
| PHKA2 | BioGrid |  |  |  |  |  |  |
| PIK3C2A | BioGrid |  |  |  |  |  |  |
| PKM | BioGrid |  |  |  |  |  |  |
| POLR1A | BioGrid |  |  |  |  |  |  |
| POLR1B | BioGrid |  |  |  |  |  |  |
| POLR1C | BioGrid |  |  |  |  |  |  |
| POLR2E | BioGrid |  |  |  |  |  |  |
| POU2F1 | BioGrid |  |  |  |  |  |  |
| PPIA | BioGrid |  | IntAct |  |  |  |  |
| PPM1A | BioGrid |  | IntAct |  |  |  |  |
| PPM1B | BioGrid |  | IntAct |  |  |  |  |
| PPP1R12A | BioGrid |  |  |  |  |  |  |
| PPP2CA |  |  |  |  | STRING |  |  |
| PPP2R1A | BioGrid |  |  |  |  |  |  |
| PPP2R4 | BioGrid |  |  |  |  |  |  |
| PRKACA |  |  |  |  |  |  | CORUM |
| PRKCB | BioGrid |  |  |  |  |  |  |
| PRMT1 | BioGrid |  |  |  |  |  |  |
| PRMT5 | BioGrid |  |  |  |  |  |  |
| PRP4B |  |  | IntAct |  |  |  |  |
| PRPF4 | BioGrid |  |  |  |  |  |  |
| PRPF4B | BioGrid |  |  |  |  |  |  |
| PTAFR | BioGrid | HPRD |  |  |  |  |  |
| PTBP1 | BioGrid |  | IntAct |  |  |  |  |
| PTGDS | BioGrid |  |  |  |  |  |  |
| PTGER2 | BioGrid |  |  |  |  |  |  |
| PTGER4 | BioGrid |  |  |  |  |  |  |
| PTGES3 | BioGrid |  |  |  |  |  |  |
| PYRG1 |  |  | IntAct |  |  |  |  |
| Q3MIH3 |  |  | IntAct |  |  |  |  |
| Q5RKT7 |  |  | IntAct |  |  |  |  |
| Q5U5U6 |  |  | IntAct |  |  |  |  |
| Q5UGI3 |  |  | IntAct |  |  |  |  |
| Q6PIH6 |  |  | IntAct |  |  |  |  |
| Q6ZSQ4 |  |  | IntAct |  |  |  |  |
| Q8TBK5 |  |  | IntAct |  |  |  |  |
| Q9NWD7 |  |  | IntAct |  |  |  |  |
| Q9UL80 |  |  | IntAct |  |  |  |  |
| RAB11FIP5 | BioGrid |  |  |  |  |  |  |
| RAB5A |  |  |  |  | STRING |  |  |
| RAB5C | BioGrid |  | IntAct |  |  |  |  |
| RAD18 | BioGrid |  |  |  |  |  |  |
| RAF1 | BioGrid | HPRD |  |  | STRING |  |  |
| RAGP1 |  |  | IntAct |  |  |  |  |
| RALGDS | BioGrid | HPRD |  |  |  |  |  |
| RAMP1 |  | HPRD |  |  |  |  |  |
| RANGAP1 | BioGrid |  |  |  |  |  |  |
| RBM10 | BioGrid |  | IntAct |  |  |  |  |
| RFA1 |  |  | IntAct |  |  |  |  |
| RFIP5 |  |  | IntAct |  |  |  |  |
| RFX1 | BioGrid |  |  |  |  |  |  |
| RHG17 |  |  | IntAct |  |  |  |  |
| RHG21 |  |  | IntAct |  |  |  |  |
| RHO | BioGrid |  |  |  |  |  |  |
| RL11 |  |  | IntAct |  |  |  |  |
| RL12 |  |  | IntAct |  |  |  |  |
| RL14 |  |  | IntAct |  |  |  |  |
| RL15 |  |  | IntAct |  |  |  |  |
| RL18 |  |  | IntAct |  |  |  |  |
| RL19 |  |  | IntAct |  |  |  |  |
| RL21 |  |  | IntAct |  |  |  |  |
| RL22 |  |  | IntAct |  |  |  |  |
| RL26 |  |  | IntAct |  |  |  |  |
| RL28 |  |  | IntAct |  |  |  |  |
| RL3 |  |  | IntAct |  |  |  |  |
| RL30 |  |  | IntAct |  |  |  |  |
| RL31 |  |  | IntAct |  |  |  |  |
| RL35 |  |  | IntAct |  |  |  |  |
| RL35A |  |  | IntAct |  |  |  |  |
| RL36 |  |  | IntAct |  |  |  |  |
| RL4 |  |  | IntAct |  |  |  |  |
| RL7 |  |  | IntAct |  |  |  |  |
| RL7A |  |  | IntAct |  |  |  |  |
| RL7L |  |  | IntAct |  |  |  |  |
| RLA0 |  |  | IntAct |  |  |  |  |
| RLA1 |  |  | IntAct |  |  |  |  |
| RLA2 |  |  | IntAct |  |  |  |  |
| RM43 |  |  | IntAct |  |  |  |  |
| RM44 |  |  | IntAct |  |  |  |  |
| ROA0 |  |  | IntAct |  |  |  |  |
| ROA1 |  |  | IntAct |  |  |  |  |
| RPA1 | BioGrid |  | IntAct |  |  |  |  |
| RPA2 |  |  | IntAct |  |  |  |  |
| RPA43 |  |  | IntAct |  |  |  |  |
| RPAB1 |  |  | IntAct |  |  |  |  |
| RPAC1 |  |  | IntAct |  |  |  |  |
| RPL11 | BioGrid |  |  |  |  |  |  |
| RPL12 | BioGrid |  |  |  |  |  |  |
| RPL14 | BioGrid |  |  |  |  |  |  |
| RPL15 | BioGrid |  |  |  |  |  |  |
| RPL18 | BioGrid |  |  |  |  |  |  |
| RPL19 | BioGrid |  |  |  |  |  |  |
| RPL21 | BioGrid |  |  |  |  |  |  |
| RPL22 | BioGrid |  |  |  |  |  |  |
| RPL26 | BioGrid |  |  |  |  |  |  |
| RPL28 | BioGrid |  |  |  |  |  |  |
| RPL3 | BioGrid |  |  |  |  |  |  |
| RPL30 | BioGrid |  |  |  |  |  |  |
| RPL31 | BioGrid |  |  |  |  |  |  |
| RPL35 | BioGrid |  |  |  |  |  |  |
| RPL35A | BioGrid |  |  |  |  |  |  |
| RPL36 | BioGrid |  |  |  |  |  |  |
| RPL4 | BioGrid |  |  |  |  |  |  |
| RPL6 | BioGrid |  |  |  |  |  |  |
| RPL7 | BioGrid |  |  |  |  |  |  |
| RPL7A | BioGrid |  |  |  |  |  |  |
| RPL7L1 | BioGrid |  |  |  |  |  |  |
| RPLP0 | BioGrid |  |  |  |  |  |  |
| RPLP1 | BioGrid |  |  |  |  |  |  |
| RPLP2 | BioGrid |  |  |  |  |  |  |
| RPN2 | BioGrid |  | IntAct |  |  |  |  |
| RPS13 | BioGrid |  |  |  |  |  |  |
| RPS17 | BioGrid |  |  |  |  |  |  |
| RPS19 | BioGrid |  |  |  |  |  |  |
| RPS27A | BioGrid |  |  |  |  |  |  |
| RPS3 | BioGrid |  |  |  |  |  |  |
| RPS3A | BioGrid |  |  |  |  |  |  |
| RPS4X | BioGrid |  |  |  |  |  |  |
| RPS6 | BioGrid |  |  |  |  |  |  |
| RPS7 | BioGrid |  |  |  |  |  |  |
| RPS8 | BioGrid |  |  |  |  |  |  |
| RS13 |  |  | IntAct |  |  |  |  |
| RS17 |  |  | IntAct |  |  |  |  |
| RS19 |  |  | IntAct |  |  |  |  |
| RS3 |  |  | IntAct |  |  |  |  |
| RS3A |  |  | IntAct |  |  |  |  |
| RS4X |  |  | IntAct |  |  |  |  |
| RS6 |  |  | IntAct |  |  |  |  |
| RS7 |  |  | IntAct |  |  |  |  |
| RS8 |  |  | IntAct |  |  |  |  |
| RTF1 | BioGrid |  |  |  |  |  |  |
| RXFP1 |  |  | IntAct | MINT |  |  | CORUM |
| S100A9 | BioGrid |  |  |  |  |  |  |
| S10A9 |  |  | IntAct |  |  |  |  |
| S1PR1 |  |  |  |  |  |  | CORUM |
| SAG | BioGrid |  |  |  |  |  |  |
| SCYL2 | BioGrid |  | IntAct |  |  |  |  |
| SDC3 | BioGrid |  | IntAct |  |  |  |  |
| SDHA | BioGrid |  | IntAct |  |  |  |  |
| SENP1 | BioGrid |  |  |  |  |  |  |
| SERBP1 | BioGrid |  |  |  |  |  |  |
| SF3B1 | BioGrid |  | IntAct |  |  |  |  |
| SF3B2 | BioGrid |  | IntAct |  |  |  |  |
| SF3B3 | BioGrid |  | IntAct |  |  |  |  |
| SFPQ | BioGrid |  | IntAct |  |  |  |  |
| SIRT1 | BioGrid |  |  |  |  |  |  |
| SLC22A5 |  | HPRD |  |  |  |  |  |
| SLC9A3R2 | BioGrid |  |  |  |  |  |  |
| SLC9A5 | BioGrid |  |  |  |  |  |  |
| SMARCC2 | BioGrid | HPRD |  |  |  |  |  |
| SMD1 |  |  | IntAct |  |  |  |  |
| SMD2 |  |  | IntAct |  |  |  |  |
| SMO |  |  | IntAct | MINT | STRING |  |  |
| SMRC2 |  |  | IntAct |  |  |  |  |
| SNRPD1 | BioGrid |  |  |  |  |  |  |
| SNRPD2 | BioGrid |  |  |  |  |  |  |
| SPIN1 | BioGrid |  | IntAct |  |  |  |  |
| SPIN3 | BioGrid |  | IntAct |  |  |  |  |
| SPNDC |  |  | IntAct |  |  |  |  |
| SPTAN1 | BioGrid |  |  |  |  |  |  |
| SPTB2 |  |  | IntAct |  |  |  |  |
| SPTBN1 | BioGrid |  |  |  |  |  |  |
| SPTN1 |  |  | IntAct |  |  |  |  |
| SRC | BioGrid |  | IntAct |  |  | DIP |  |
| SRC8 |  |  | IntAct |  |  |  |  |
| SRPK2 | BioGrid |  | IntAct |  |  |  |  |
| SRRM2 | BioGrid |  | IntAct |  |  |  |  |
| STAT1 | BioGrid |  | IntAct |  |  |  |  |
| STC2 | BioGrid | HPRD | IntAct |  |  |  |  |
| STK38 | BioGrid |  | IntAct |  |  |  |  |
| STUB1 | BioGrid |  |  |  |  |  |  |
| SYNE2 | BioGrid |  | IntAct |  |  |  |  |
| TAB1 | BioGrid |  | IntAct |  |  |  |  |
| TAB2 | BioGrid |  |  |  |  |  |  |
| TACR1 |  |  |  |  | STRING |  |  |
| TBA1A |  |  | IntAct |  |  |  |  |
| TBA1C |  |  | IntAct |  |  |  |  |
| TBA3C |  |  | IntAct |  |  |  |  |
| TBA4A |  |  | IntAct |  |  |  |  |
| TBA8 |  |  | IntAct |  |  |  |  |
| TBB2A |  |  | IntAct |  |  |  |  |
| TBB3 |  |  | IntAct |  |  |  |  |
| TBB4A |  |  | IntAct |  |  |  |  |
| TBB4B |  |  | IntAct |  |  |  |  |
| TBXA2R | BioGrid |  |  |  | STRING |  |  |
| TCOF |  |  | IntAct |  |  |  |  |
| TCOF1 | BioGrid |  |  |  |  |  |  |
| TCPZ |  |  | IntAct |  |  |  |  |
| TERA |  |  | IntAct |  |  |  |  |
| TFAP4 | BioGrid |  |  |  |  |  |  |
| TGFBR3 |  | HPRD |  |  | STRING |  |  |
| THOC4 |  |  | IntAct |  |  |  |  |
| THRAP3 | BioGrid |  |  |  |  |  |  |
| TIF1B |  |  | IntAct |  |  |  |  |
| TMEM169 | BioGrid |  |  |  |  |  |  |
| TMEM38A | BioGrid |  |  |  |  |  |  |
| TMOD3 | BioGrid |  | IntAct |  |  |  |  |
| TMPO | BioGrid |  |  |  |  |  |  |
| TNFSF8 | BioGrid |  |  |  |  |  |  |
| TPCN2 | BioGrid |  |  |  |  |  |  |
| TR150 |  |  | IntAct |  |  |  |  |
| TRAF6 | BioGrid |  | IntAct |  |  | DIP |  |
| TRHR | BioGrid |  |  |  |  |  |  |
| TRIM28 | BioGrid |  |  |  |  |  |  |
| TRPV4 | BioGrid |  | IntAct |  |  |  |  |
| TUBA1A | BioGrid |  |  |  |  |  |  |
| TUBA1C | BioGrid |  |  |  |  |  |  |
| TUBA3C | BioGrid |  |  |  |  |  |  |
| TUBA4A | BioGrid |  |  |  |  |  |  |
| TUBA8 | BioGrid |  |  |  |  |  |  |
| TUBB2A | BioGrid |  |  |  |  |  |  |
| TUBB3 | BioGrid |  |  |  |  |  |  |
| TUBB4A | BioGrid |  |  |  |  |  |  |
| TUBB4B | BioGrid |  |  |  |  |  |  |
| TWISTNB | BioGrid |  |  |  |  |  |  |
| U5S1 |  |  | IntAct |  |  |  |  |
| UBA52 | BioGrid |  |  |  |  |  |  |
| UBB | BioGrid |  |  |  |  |  |  |
| UBC | BioGrid |  |  |  | STRING |  |  |
| UBR5 | BioGrid |  | IntAct |  |  |  |  |
| USP33 | BioGrid |  |  |  | STRING |  |  |
| VIM | BioGrid |  |  |  |  |  |  |
| VIME |  |  | IntAct |  |  |  |  |
| VPS35 |  |  | IntAct |  |  |  |  |
| WDR26 |  |  | IntAct |  |  |  |  |
| WDR77 | BioGrid |  |  |  |  |  |  |
| WEE1 | BioGrid |  | IntAct |  |  |  |  |
| WNT5A |  |  |  |  | STRING |  |  |
| XPO1 | BioGrid |  |  |  |  |  |  |
| XRCC5 |  |  | IntAct |  |  |  |  |
| XRCC6 |  |  | IntAct |  |  |  |  |
| YBOX1 |  |  | IntAct |  |  |  |  |
| YBOX3 |  |  | IntAct |  |  |  |  |
| YWHAB |  |  |  |  |  |  |  |
| YWHAE |  |  |  |  |  |  |  |
| YWHAG |  |  |  |  |  |  |  |
| YWHAH |  |  |  |  |  |  |  |
| YWHAQ | BioGrid |  | IntAct |  |  |  |  |
| ZRAB2 |  |  | IntAct |  |  |  |  |
| ZRANB2 | BioGrid |  |  |  |  |  |  |

**Table S3: *Interrogation of interactome metadata for β-arrestin1 and 2 using GeneIndexer***

|  | **ARRB1 - Aging** | | | | | | | | | |
| --- | --- | --- | --- | --- | --- | --- | --- | --- | --- | --- |
| **Gene** | *H1FX* | *WDR77* | *IGHMBP2* | *GPR50* | *HIBCH* | *NOP10* | *ZBTB43* | *THRAP3* | *EEF1A2* | *BCLAF1* |
| *Ageing* | 0.163 | 0.153 | 0.134 | 0.178 | 0.124 | 0.187 | 0.107 | 0.192 | 0.206 | 0.396 |
| *Aging* | 0.196 | 0.182 | 0.222 | 0.16 | 0.258 | 0.225 | 0.151 | 0.213 | 0.231 | 0.349 |
| *Senescence* | 0.176 | 0.166 | 0 | 0 | 0 | 0.296 | 0.129 | 0.185 | 0.105 | 0.218 |
| *Senescent* | 0 | 0 | 0 | 0 | 0 | 0 | 0 | 0 | 0 | 0 |
| *Elderly* | 0 | 0 | 0.134 | 0.137 | 0.168 | 0 | 0.206 | 0 | 0.111 | 0 |
| *Elder* | 0 | 0 | 0.118 | 0.152 | 0.135 | 0 | 0.138 | 0 | 0.135 | 0 |
| *Longevity* | 0 | 0.101 | 0 | 0 | 0 | 0 | 0 | 0.175 | 0 | 0.172 |
| **Cosine Sum** | 0.535 | 0.602 | 0.608 | 0.627 | 0.685 | 0.708 | 0.731 | 0.765 | 0.788 | 1.135 |
|  |  |  |  |  |  |  |  |  |  |  |
|  | **ARRB2 - Aging** | | | | | | | | | |
| **Gene** | *SIRT1* | *BCLAF1* | *MRPL43* | *DKC1* | *THRAP3* | *EEF1A2* | *ANKRD11* | *WDR77* | *HNRNPC* | *EEF2* |
| *Ageing* | 0.469 | 0.396 | 0.202 | 0.188 | 0.192 | 0.206 | 0.178 | 0.153 | 0.14 | 0.101 |
| *Aging* | 0.532 | 0.349 | 0.293 | 0.213 | 0.213 | 0.231 | 0.162 | 0.182 | 0.143 | 0.162 |
| *Senescence* | 0.255 | 0.218 | 0.139 | 0.336 | 0.185 | 0.105 | 0.249 | 0.166 | 0.105 | 0.101 |
| *Senescent* | 0.19 | 0.153 | 0.145 | 0.239 | 0.178 | 0.104 | 0.152 | 0.156 | 0.114 | 0.104 |
| *Elderly* | 0 | 0 | 0.1 | 0 | 0 | 0.111 | 0 | 0 | 0 | 0 |
| *Elder* | 0 | 0 | 0.107 | 0 | 0 | 0.135 | 0 | 0 | 0 | 0 |
| *Longevity* | 0.676 | 0.172 | 0 | 0 | 0.175 | 0 | 0.139 | 0.101 | 0 | 0 |
| **Cosine Sum** | 2.122 | 1.288 | 0.986 | 0.976 | 0.943 | 0.892 | 0.88 | 0.758 | 0.502 | 0.468 |

**
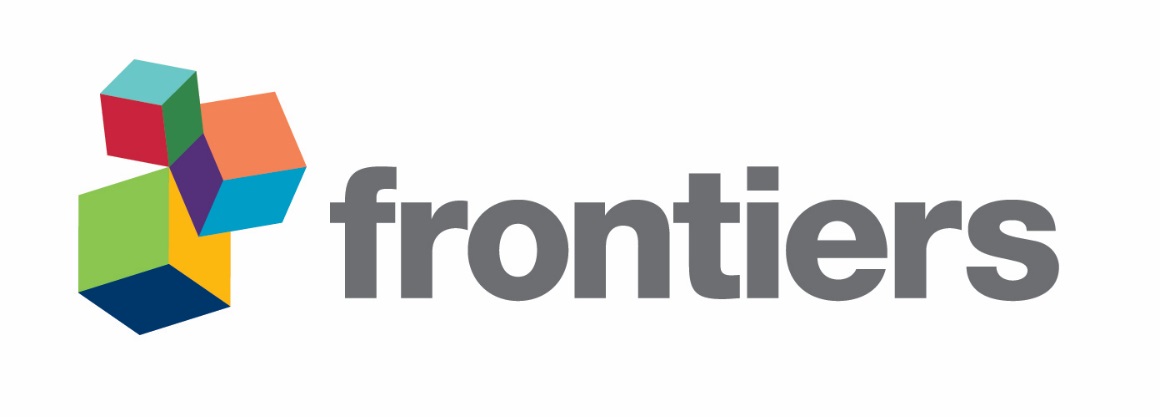
**
